# Transient cardiomyocyte fusion regulates cardiac development in zebrafish

**DOI:** 10.1038/s41467-017-01555-8

**Published:** 2017-11-15

**Authors:** Suphansa Sawamiphak, Zacharias Kontarakis, Alessandro Filosa, Sven Reischauer, Didier Y. R. Stainier

**Affiliations:** 1 Department of Developmental Genetics, Max Planck Institute for Heart and Lung Research, Bad Nauheim, 61231 Germany; 2 Max Delbrück Center for Molecular Medicine, Robert-Rössle-Straße 10, 13092 Berlin, Germany; 3 Present Address: Max Delbrück Center for Molecular Medicine, Robert-Rössle-Straße 10, 13092 Berlin, Germany; DZHK (German Center for Cardiovascular Research), Partner Site Berlin, Berlin, Germany

## Abstract

Cells can sacrifice their individuality by fusing, but the prevalence and significance of this process are poorly understood. To approach these questions, here we generate transgenic reporter lines in zebrafish to label and specifically ablate fused cells. In addition to skeletal muscle cells, the reporters label cardiomyocytes starting at an early developmental stage. Genetic mosaics generated by cell transplantation show cardiomyocytes expressing both donor- and host-derived transgenes, confirming the occurrence of fusion in larval hearts. These fusion events are transient and do not generate multinucleated cardiomyocytes. Functionally, cardiomyocyte fusion correlates with their mitotic activity during development as well as during regeneration in adult animals. By analyzing the cell fusion-compromised *jam3b* mutants, we propose a role for membrane fusion in cardiomyocyte proliferation and cardiac function. Together, our findings uncover the previously unrecognized process of transient cardiomyocyte fusion and identify its potential role in cardiac development and function.

## Abstract (web summary)

Cell fusion regulates several physiological events, for example, fusion of myoblasts in skeletal muscle formation, but it is unclear if this process occurs in the heart. Here, the authors use transgenic reporters in zebrafish to show transient cardiomyocyte fusion, modulating cardiac development and function.

## Introduction

Cell fusion is an indispensable process in diverse physiological and pathological events. Fusion of myoblasts into multinucleated syncytia is fundamental to skeletal myogenesis in most organisms^1^. Myeloid lineage cells also undergo homotypic fusion to generate bone-resorbing osteoclasts and chronic inflammatory giant cells^2^. Cancer cell fusion with bone marrow-derived cells has been proposed to serve as a mechanism of metastasis and generation of cancer stem cells^3^. Moreover, the concept of fusion-mediated reprogramming of differentiated cells is well established^4–6^. Although cell fusion is involved in a wide range of cellular processes and holds therapeutic promise, our understanding of the underlying mechanisms and significance of cell fusion remains limited, mainly due to the lack of experimental models that allow in vivo visualization of fusion events. Fusion-mediated cell fate reprogramming, observed in stem-somatic cell heterokaryons, could aid tissue regeneration through its potential to revert the somatic cell differentiated state and restore embryonic self-renewal capacity^5^. Another example of the therapeutic potential of cell fusion relates to the reversal of altered phenotypes through fusion-mediated complementation of recessive mutations using wild-type cells, as shown in the liver^7, 8^. Mammalian cardiomyocytes have long been considered terminally differentiated in association with cell cycle arrest during the maturation of the myocardium^9^. The ability to stimulate mature cardiomyocyte de-differentiation and cell cycle re-entry, as likely occurring in the adult zebrafish heart after injury^10, 11^, has been a primary goal in regenerative medicine. While fusion between cardiomyocytes and bone marrow-derived progenitor cells contributes minimally to the generation of new cardiomyocytes in the injured mouse heart^12^, the role of cell fusion in cardiac regeneration is yet to be explored.

In vivo assessment of cell fusion has thus far primarily relied on transplanting specific cell types, genetically marked, into unmarked or differentially marked host animals^13–15^. While providing essential information about the fusion competency of the cell types under study, these transplantation methods cannot identify unknown fusion events. Sporadic fusion between cardiomyocytes and circulating cells, while rarely occurs, have been consistently reported in healthy^13^ and infarcted^14, 15^ myocardium following bone marrow transplantation of sublethally irradiated mice. The earlier observation of somatic-to-embryonic stem cell reprogramming upon fusion^5^ leads to the hypothesis that fusion with blood progenitors might be able to drive post-mitotic cardiomyocytes to proliferation^15, 16^. However, the low frequency of these fusion events has hindered the assessment of their potential benefit^14, 16^. To facilitate ubiquitous detection of cell fusion in vivo, we developed transgenic tools that utilize differential Cre recombination to generate mosaic cell populations expressing either a GAL4 driver or a UAS based reporter. We show that these tools successfully label fusion-derived cells. Using these tools and genetic mosaics generated by transplantation, we uncover a previously unrecognized fusion process that allows transient cytoplasmic connections between cardiomyocytes in zebrafish. Analysis of the fusion-derived cardiomyocyte population employing our newly developed transgenic system reveals that the occurrence of fusion correlates with their mitotic activity during larval growth as well as after injury in adults. Correspondingly, analysis of a cell fusion-deficient mutant shows that membrane fusion positively modulates cardiomyocyte proliferation and cardiac contractility. In summary, we report here frequent cardiomyocyte fusion events that occur during development and regeneration of the zebrafish heart, as well as their unappreciated role in myocardial growth and function.

## Results

### Generation of the FATC transgenic line

**Fig. 1:**
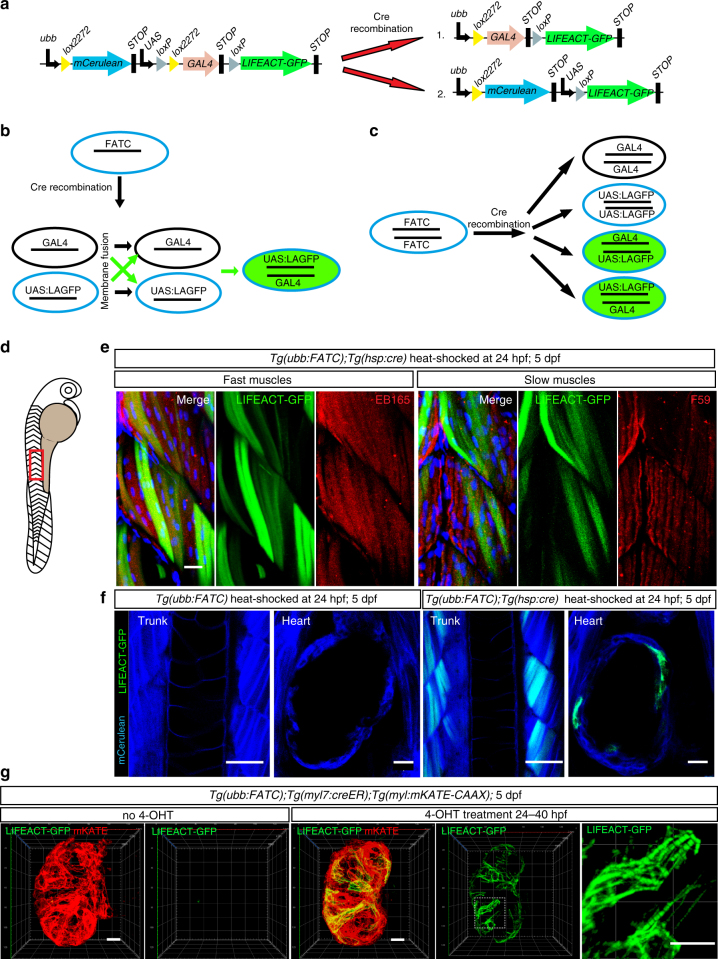
Establishment of the FATC transgenic line for in vivo labeling of fusion-derived cells. **a** Schematic illustration of the *ubb:FATC* construct and Cre recombination products. **b**, **c** Schematic illustrations of membrane fusion (**b**) and multiple transgene insertions (**c**) that could give rise to LIFEACT-GFP expression. **b** Cre recombination of *Tg(ubb:FATC)* animals harboring a single copy of the transgene generates two different cell populations, one carrying a *GAL4* expression cassette and the other carrying a *UAS:LIFEACT-GFP* (LAGFP) cassette. Fusion between cells carrying different cassettes (green arrows), but not between cells carrying the same cassette (black arrows), leads to activation of LIFEACT-GFP expression (green). **c** Stochastic Cre recombination in cells harboring two copies of the *FATC* transgene will generate 50% of the time cells carrying both the *GAL4* and *UAS:LIFEACT-GFP* cassettes (green). **d**, **e** LIFEACT-GFP expression is activated in fast twitch, but excluded from slow twitch, muscles. **d** Drawing of a zebrafish larva depicting the trunk area (red box), shown in **e**. **e** *Tg(ubb:FATC);Tg(hsp:cre)* embryos were heat-shocked at 24 hpf, and LIFEACT-GFP expression (green) was assessed at 5 dpf. Muscle fibers were visualized by immunostaining with EB165 and F59 (red) to label fast and slow twitch muscles, respectively. DAPI (blue) labeling shows fusion-derived multinucleation of fast twitch muscles. **f** heat induction of *cre* expression at 24 hpf activates LIFEACT-GFP (green) expression in skeletal and cardiac myocytes in 5 dpf *Tg(ubb:FATC*);*Tg(hsp:cre)* larvae, but not in *Tg(ubb:FATC)* animals (blue). **g** LIFEACT-GFP expression (green) labels sarcomeric structures of FATC-activated cardiomyocytes (identifiable by membrane expression of mKATE-CAAX (red)). Absence of LIFEACT-GFP expression without 4-OHT treatment confirms the dependency of the FATC reporter on Cre activity. **e**, **f** show maximum intensity projections of 10–50 μm thick confocal stacks. **g** 3D volume renderings of 90 μm thick confocal stacks of the entire cardiac ventricle. Representative images from a total of 8–9 (**e**), 32 (**f**), and 9 (**g**) animals are shown. Scale bars: 20 μm (**e**, **f** trunk, **g**), 50 μm (**f** heart)

The best known cell–cell fusion events are the complete merging of cell membranes to generate multinucleated or polyploid cells^1, 17^^,^. Additionally, fusion of cell membranes can occur in only parts of the cell boundaries and lead to transient cytoplasmic exchange^18, 19^. To distinguish between these events, we refer to partial or transient fusion as “membrane fusion”, and complete fusion as “cell fusion”. To identify unknown fusion events, we generated a transgenic line, *Tg(ubb:lox2272-mCerulean-UAS-loxP-lox2272-GAL4-loxP-LIFEACT-GFP)* ^*bns97*^, that can report both types of fusion (Fig. 1a). Because of the mutual incompatibility of loxP and lox2272 sites^20, 21^, only one recombination event will take place per transgene. Therefore, Cre recombination stochastically leads to *ubb:lox2272-GAL4-loxP-LIFEACT-GFP* or *ubb:lox2272-mCerulean-UAS-loxP-LIFEACT-GFP*. When these two transgenes come together, e.g., after membrane or cell fusion, GAL4 will bind the UAS sequence and activate *LIFEACT-GFP* expression (Fig. 1b). We thus named this transgenic system “Fluorescence Activation after Transgene Coupling” (FATC). Suitability of a FATC line to label exclusively cells that have undergone fusion depends on the transgene copy number, i.e., a FATC line containing multiple transgene insertions is expected to exhibit widespread *LIFEACT-GFP* expression following Cre recombination (Fig. 1c). Thus, we first assessed the number of transgene insertions in the *Tg(ubb:FATC)* ^*bns97*^ line. *Tg(ubb:FATC)* ^*bns97*^ outcrosses generated 50% of offspring that carried the FATC transgene, as assessed by mCerulean expression, suggesting a single transgene insertion. Moreover, heat-induced ubiquitous expression of *cre* in *Tg(ubb:FATC)* ^*bns97*^ *;Tg*(*hsp:cre)* embryos at 24 hours post fertilization (hpf) resulted in transgene-coupling activation of LIFEACT-GFP expression in only a few tissues (Supplementary Fig. 1). To test the ability of the *bns97* FATC line to label fusion-derived cells specifically, we examined LIFEACT-GFP expression in distinct populations of skeletal myocytes. During vertebrate myogenesis, fast twitch muscles undergo extensive fusion to become multinucleated syncytia, whereas slow twitch muscles remain mononucleated^22^. Following heat-activation of Cre expression at 24 hpf, 5 days post fertilization (dpf) *Tg(ubb:FATC)* ^*bns97*^ *;Tg*(*hsp:cre)* larvae showed restricted LIFEACT-GFP expression in the cell fusion-derived multinucleated fast twitch muscles, but not in the fusion-incompetent slow twitch muscles (Fig. 1d, e), which are located more superficially. Besides the skeletal tissue, LIFEACT-GFP^+^ cells were observed in the skin, a tissue commonly reported to become polyploid^23, 24^ (Supplementary Fig. 1A). In agreement with the restricted pattern of LIFEACT-GFP expression in these animals, we found that three linked *ubb:FATC* insertions were resolved into a single copy following Cre recombination, suggesting tandem insertion of the transgene in the *Tg(ubb:FATC)* ^*bns97*^ line (Supplementary Fig. 2).

### FATC labels cardiomyocytes in the developing hearts

In addition to LIFEACT-GFP^+^ cells in skeletal muscle (Fig. 1e, f) and skin, we unexpectedly observed a substantial number of LIFEACT-GFP^+^ cells in the heart (Fig. 1f) after ubiquitous Cre expression. By virtue of their location within the developing myocardium, we posited that these cells were cardiomyocytes. To verify the identity of these cells, we assessed FATC reporter expression in *Tg(ubb:FATC);Tg(myl7:creER);Tg(myl7:mKATE-CAAX)* larvae (Fig. 1g). *myl7:creER* enables cardiomyocyte-specific expression of *creER*, which was activated by a 16 h treatment of 4-OHT starting at 24 hpf. Recombination efficiency of the same 4-OHT treatment paradigm was tested in *Tg(loxP-DsRed-loxP-EGFP);Tg(myl7:creER)* embryos. Recombination-derived EGFP^+^ cardiomyocytes populated ~74% of the total cardiac ventricle area (Supplementary Fig. 3A, B). Expression of EGFP was restricted to the heart, confirming the specific expression of *creER* in the myocardium (Supplementary Fig. 3D). Without 4-OHT treatment, *Tg(ubb:FATC);Tg(myl7:creER);Tg(myl7:mKATE-CAAX)* larvae exhibited no LIFEACT-GFP expression (Fig. 1g), indicating no leakiness of the Cre/lox system with these transgenic lines. Following CreER activation in the myocardium, a substantial number of LIFEACT-GFP^+^ cardiomyocytes, comparable to that observed upon heat induction to achieve ubiquitous *cre* expression (Fig. 1f), were detectable at 5 dpf (Fig. 1g). Membrane-localized mKATE-CAAX marked all cardiomyocytes. Importantly, LIFEACT-GFP binding to F-actin^25^ showed the presence of myofibrillar arrays in all FATC-activated cells, further confirming their cardiomyocyte identity (Fig. 1g).

### Cardiomyocyte mitosis does not activate FATC

**Fig. 2:**
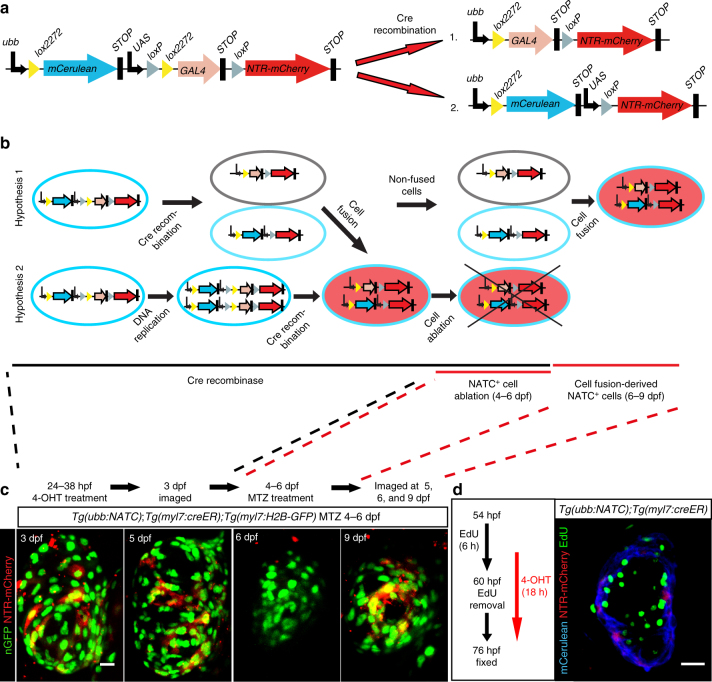
Cardiomyocyte proliferation does not mediate FATC reporter expression. **a** Schematic illustration of the *ubb:NATC* construct and Cre recombination products. **b** Schematic illustration of the cellular events that could occur in the experiment shown in **c**. **c** NTR-mCherry^+^ (red) cardiomyocytes (H2B-GFP^+^, green) were observed in *Tg(ubb:NATC);Tg(myl7:creER);Tg(myl7:H2B-GFP)* larvae following MTZ-mediated cell ablation, 7 days after transient CreER activation by 4-OHT treatment at 24 hpf. All images show the same heart. **d** NTR-mCherry expression does not succeed cardiomyocyte DNA synthesis. The 6 h EdU pulse-labeled cells undergoing DNA synthesis (green) in 54 hpf *Tg(ubb:NATC);Tg(myl7:creER)* embryos. Two hours prior to EdU removal, Cre activity was induced by an 18 h 4-OHT treatment. mCerulean (blue) and NTR-mCherry (red) expression was visualized by immunostaining. No EdU^+^NTR-mCherry^+^ cardiomyocytes were detectable in 20 hearts imaged. **c**, **d** Maximum intensity projections of 40–60 μm thick confocal stacks. Representative images from a total of 12 (**c**) and 20 (**d**) animals are shown. Scale bars: 10 μm (**c**), 20 μm (**d**)

Mammalian cardiomyocytes can become polyploid due to abortive cytokinesis^26, 27^. Besides membrane or cell fusion, Cre activity during the polyploid state following DNA replication and preceding cytokinesis, might be able to induce LIFEACT-GFP expression. To test whether mitosis-derived polyploidy accounts for the presence of LIFEACT-GFP^+^ cardiomyocytes, we generated a Nitroreductase Activation after Transgene Coupling (NATC) construct (Fig. 2a) by replacing the *LIFEACT-GFP* cassette in the *ubb:FATC* construct with a cassette encoding *mCherry* fused to nitroreductase (NTR). NTR converts the innocuous drug metronidazole (MTZ) into a cytotoxic molecule, killing the cells expressing the enzyme^28, 29^. We generated the NATC line *Tg(ubb:NATC)* ^*bns98*^, which harbors a single insertion of the transgene (Supplementary Fig. 2B, C). NATC reporter expression following ubiquitous *cre* expression generated NTR-mCherry^+^ cells in specific tissues phenocopying the behavior of the *Tg(ubb:FATC)* ^*bns97*^ line (Supplementary Fig. 4), suggesting that both lines are suitable reporters of membrane or cell fusion.

Analysis of NTR-mCherry expression enabled the distinction between NATC-activated cells derived from membrane/cell fusion events (Fig. 2b, hypothesis 1) and those derived from abortive cytokinesis (Fig. 2b, hypothesis 2). More specifically, *myl7*:CreER activation from at 24 to 38 hpf was used to mark cardiomyocytes generated by both processes, fusion and mitosis. These cardiomyocytes were subsequently ablated by MTZ treatment from 4 to 6 dpf. Thus, NATC activation at a later time point, when CreER was most likely no longer active (Supplementary Fig. 5) could only occur through membrane or cell fusion. Indeed, in the same hearts in which cardiomyocytes turning on NTR-mCherry expression before 6 dpf were ablated, we observed re-appearance of NTR-mCherry^+^ cells at 9 dpf (Fig. 2c), suggesting that membrane or cell fusion activates NATC in the larval heart. In addition, in order to detect mitosis-derived NTR-mCherry^+^ cells, we labeled newly synthesized DNA in 54 hpf *Tg(ubb:NATC)* ^*bns98*^ *;Tg(myl7:creER)* embryos with a 6 h pulse of 5-ethynyl-2’-deoxyuridine (EdU) prior to CreER activation by 4-OHT treatment from 58 to 76 hpf, thereby allowing a 2 h interval during which cardiomyocytes were exposed to both 4-OHT and EdU (Fig. 2d). If fusion had occurred during cell cycling, EdU^+^NTR-mCherry^+^ cardiomyocytes should be present. However, all detectable NTR-mCherry^+^ cardiomyocytes in 76 hpf larvae lacked EdU incorporation, indicating that NATC activation does not occur during cell division (Fig. 2d). Together, these findings suggest that the expression of NTR-mCherry in the myocardium is not due to Cre activity during cell cycling or due to cytokinesis delay/failure, but reports cardiomyocyte fusion.

### Homotypic cardiomyocyte membrane fusion

**Fig. 3:**
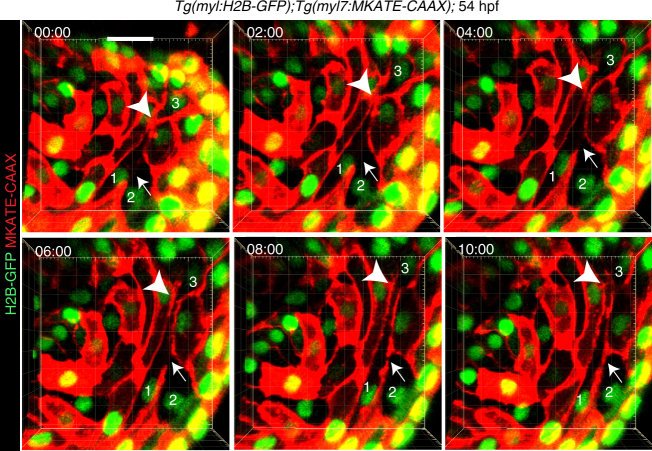
Membrane fusion occurs in the developing heart. Live imaging of *Tg(myl7:MKATE*-*CAAX);Tg(myl7:H2B-GFP)* embryos, in which heartbeats were blocked by morpholino-mediated *tnnt2* knock-down (Supplementary Movie 1), showing establishment of a new membrane border (arrows) between cardiomyocytes 1 and 2, which initially exhibited cytoplasmic continuum. The plasma membrane border between cardiomyocytes 1 and 3 (arrowheads) dissolved and was subsequently re-established. mKATE-CAAX (red) and H2B-GFP (green) expression labeled cardiomyocyte membranes and nuclei, respectively. Timing of each still image is hour:minute. All images are 3D volume renderings of 60 μm thick confocal stacks of a representative heart showing the myocardial monolayer observed from the lumen. Examples of cytoplasmic continuum between cardiomyocytes were observed in all 6 hearts examined. Scale bar: 20 μm

Thus far, only the ability of cardiomyocytes to undergo heterotypic fusion with other cell types has been reported^13–15^. Homotypic or heterotypic fusion could give rise to F/NATC activation following ubiquitous *cre* expression (Fig. 1f). By contrast, cardiomyocyte-specific *myl7:creER* expression should label mainly homotypic fusion-derived cells (Figs. 1g and 2c). However, remaining CreER activity after heterotypic fusion of cardiomyocytes with other cells might be able to activate F/NATC. To directly visualize the occurrence of cardiomyocyte fusion events in the developing heart, we performed time-lapse imaging of 54 hpf embryos in which cardiomyocytes were labeled by membrane mKATE-CAAX and nuclear H2B-GFP expression. Heartbeat in these embryos was blocked by morpholino-mediated knockdown of the *tnnt2* gene^30^, which encodes cardiac muscle troponin T. Interestingly, 16 h long live imaging captured several events of localized cell membrane fusion between neighboring cardiomyocytes, which allowed cytoplasmic connections between them. These events were only transient as new membranes were established shortly afterwards (Fig. 3; Supplementary Movie 1). The observed dissolving and/or re-establishment of plasma membranes might occur during different cellular processes including membrane rupture, completion of cytokinesis, and membrane fusion. Membrane rupture is unlikely to occur since it would lead to the direct connection of intra- and extra-cellular environments resulting in drastic intracellular changes and most likely cell death. Completion of cytokinesis in cycling cardiomyocytes might be able to explain some of the observed events (for example see arrow between cardiomyocytes 1 and 2 in Fig. 3). However, the dissolving and re-establishment of cell membranes at the interface of other adjacent cardiomyocytes (for example see arrowhead between cardiomyocytes 1 and 3 in Fig. 3) are most likely caused by transient membrane fusion events. Consistent with this interpretation, our previous observations of the NATC reporter (Fig. 2c) indicate that cardiomyocyte fusion does not occur during mitosis. Thus, our findings provide evidence for the occurrence of transient membrane fusion between cardiomyocytes.

### Cardiomyocyte fusion is detectable by cell transplantation

**Fig. 4:**
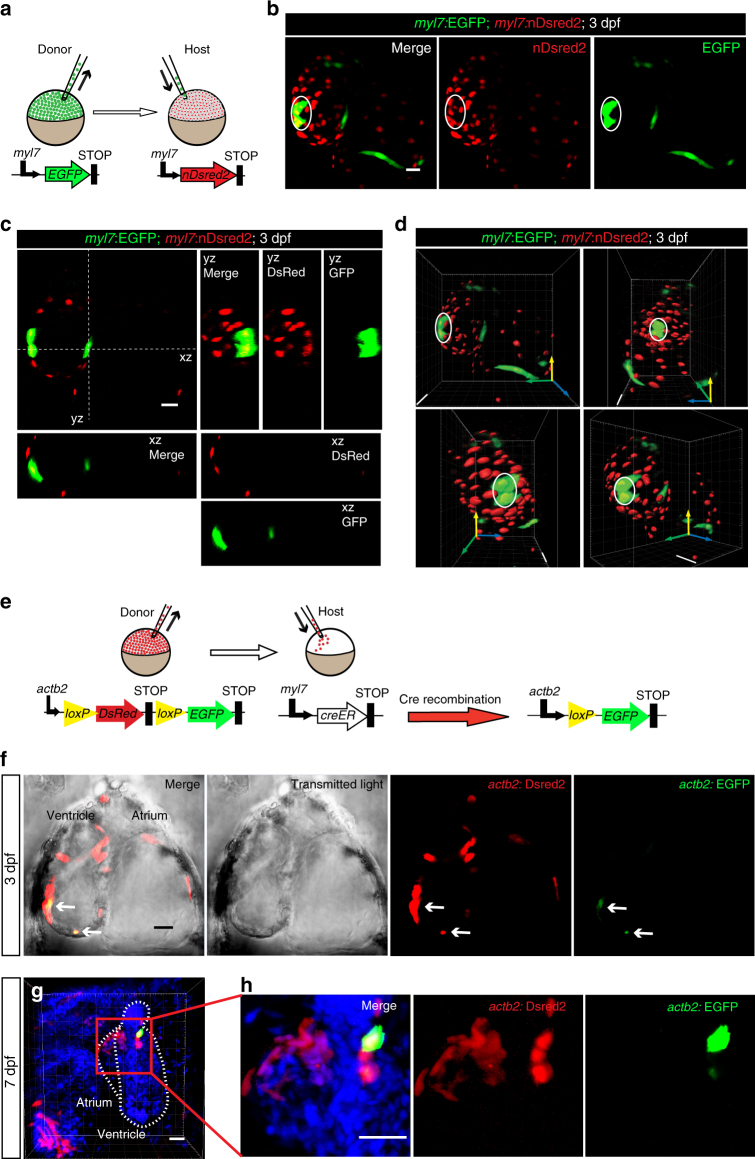
Blastula transplantations reveal cardiomyocytes expressing both donor and host transgenes. **a** Schematic drawing of transplantation experiment shown in **b**–**d**. *Tg(myl7:EGFP)* cells were transplanted into *Tg(myl7:nDsred2)* hosts at the blastula stage. **b**–**d** Cardiomyocytes expressing both host-derived *myl7*:EGFP (green cytoplasm) and donor-derived *myl7:*nDsred2 (red nucleus) transgenes (**b**) are evident from orthogonal sections (**c**) and *Y* axis rotation of a 3D volume rendering (**d**). EGFP^+^nDsred2^+^ cardiomyocytes (white circle) were detected in 9 out of 18 mosaic hearts at 3 dpf. **e** Schematic drawing of transplantation experiment shown in **f**–**h**. *Tg(actb2:loxP-mCherry-loxP-EGFP)* cells were transplanted into *Tg(myl7:creER)* hosts at the blastula stage. Transplanted animals were treated with 4-OHT starting at 24 hpf. New 4-OHT was added to the embryo medium daily. **f** A single focal plane of a confocal stack shows a dorsal view of a 3 dpf chimeric heart, anterior up. Donor-derived mCherry^+^ cells (red) and fusion-derived EGFP^+^ cardiomyocytes (green, arrows) were detected by live imaging in 5 out of 13 mosaic hearts (mixed genotypes, creER^+^ and creER^−^). **g** Maximum intensity projection of a 50 μm confocal stack shows a lateral view of a 7 dpf chimeric heart, anterior up, dorsal to the left. *Tg(actb2:loxP-mCherry-loxP-EGFP)* donor cells are shown in red. Host-derived Cre-mediated recombination of the donor transgene was detectable by EGFP immunofluorescence (green) in 11 out of 39 mosaic hearts (mixed genotypes, creER^+^, and creER^−^). **h** Magnified image showing area outlined by red box in **g**. Scale bars: 20 μm

To further test the presence of membrane or cell fusion in developing cardiomyocytes, we used a transplantation approach to create mosaic animals. Cells from *Tg(myl7:EGFP)* blastulae were transplanted into *Tg(myl7:nDsRed2)* blastulae (Fig. 4a). In 9 out of 18 mosaic hearts, we observed cardiomyocytes expressing both donor- and host-derived transgenes at 3 dpf (Fig. 4b–d). Orthogonal sections (Fig. 4c) and 3D surface renderings (Fig. 4d) confirmed the presence of cytoplasmic EGFP and nuclear DsRed2 in these cardiomyocytes. We also used *Tg(actb2:loxP-DsRed-loxP-EGFP)* embryos as donors and *Tg(myl7:creER)* embryos as hosts (Fig. 4e). In this case, fusion would lead to EGFP expression after CreER-mediated recombination in cardiomyocytes. Following daily 4-OHT treatment starting at 24 hpf, EGFP^+^ cells were detectable in 5 out of 13 mosaic hearts at 3 dpf (Fig. 4f). These EGFP^+^ cells were localized in the single-layered compact wall or in the trabeculae, suggesting their cardiomyocyte identity. At later developmental stages, between 5 and 7 dpf, we were also able to detect EGFP^+^ cardiomyocytes in 11 out of 39 mosaic hearts (Fig. 4g, h). Together with our observations on *Tg(ubb:FATC)* ^*bns97*^ and *Tg(ubb:NATC)* ^*bns98*^ animals, data from these two transplantation-based strategies provide strong evidence for fusion events that lead to the formation of cytosolic connections and allow the exchange of mRNAs and/or proteins between cardiomyocytes.

### F/NATC labels a proliferative pool of cardiomyocytes

**Fig. 5:**
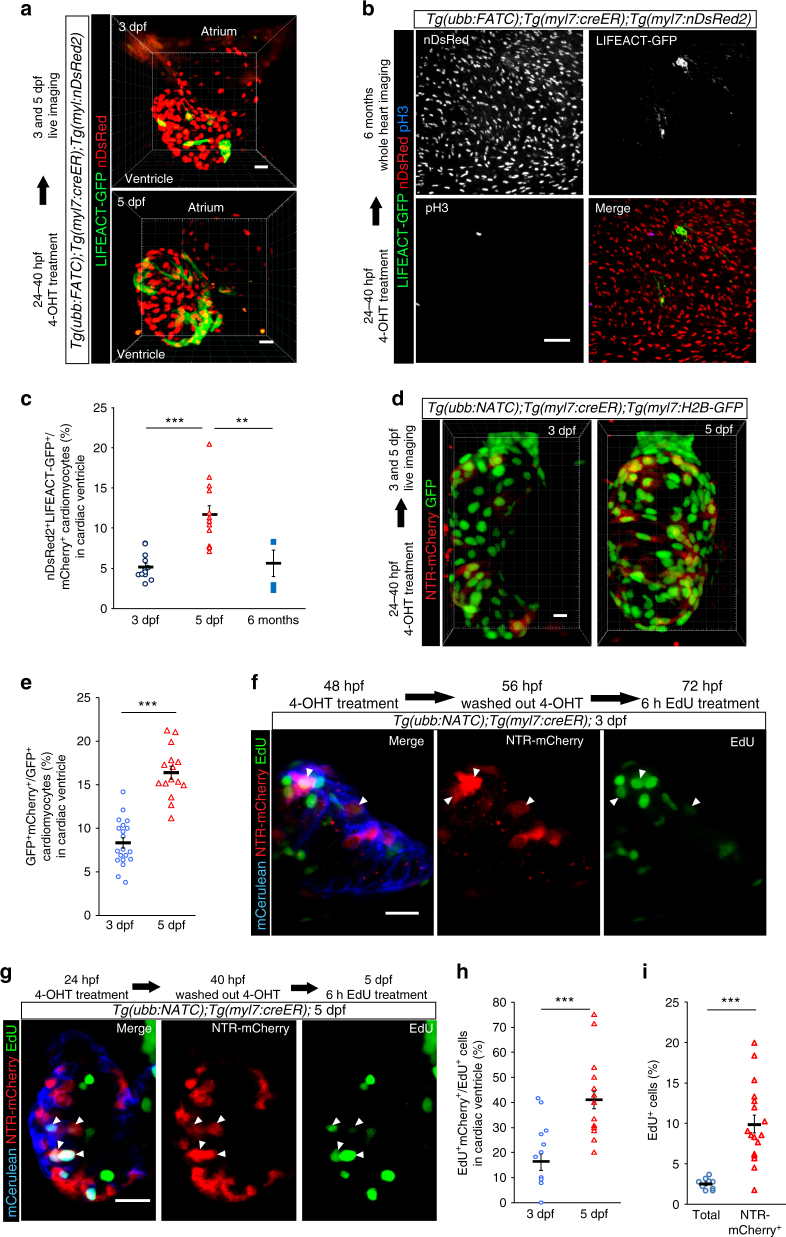
F/NATC-labeled cardiomyocytes are highly proliferative. **a**–**c** FATC-activated (LIFEACT-GFP^+^, green) cardiomyocytes (nDsRed^+^, red) in 3 and 5 dpf (**a**) and 6 mpf (**b**) *Tg(ubb:FATC);Tg(myl7:creER);Tg(myl7:nDsRed2)* fish treated with 4-OHT from 24 to 40 hpf were quantified as percentages of total ventricular cardiomyocytes (**c**). The same fish were analyzed at 3 and 5 dpf (**a**, **c**). **d**, **e** NTR-mCherry^+^ (red) cardiomyocytes (H2B-GFP^+^, green) of 3 and 5 dpf *Tg(ubb:NATC);Tg(myl7:creER);Tg(myl7:H2B-GFP)* fish treated with 4-OHT starting at 24 hpf (**d**) were quantified as percentage of total ventricular cardiomyocytes (**e**). **f**–**h** NTR-mCherry^+^ cardiomyocytes contribute substantially to the proliferating subset of cardiomyocytes. *Tg(ubb:NATC);Tg(myl7:creER)* embryos were treated with 4-OHT starting at 48 (**f**) or 24 (**g**) hpf to identify NATC-activated cardiomyocytes (mCerulean^+^,blue, and mCherry^+^, red). A 6 h EdU pulse (green) starting at 72 (**f**) or 120 (**g**) hpf labeled cells undergoing DNA synthesis. Arrowheads point to mCerulean^+^mCherry^+^EdU^+^ cardiomyocytes, which were quantified as percentages of total EdU^+^ ventricular cardiomyocytes (**h**). **i** Percentages of proliferating cardiomyocytes, assessed by a 6 h pulse of EdU, relative to total cardiomyocytes and relative to the NTR-mCherry^+^ cardiomyocyte population in 5 dpf *Tg(myl7:nuDsRed2)* and *Tg(myl7:creER);Tg(ubb:NATC)* ventricles, respectively. Three-dimensional volume renderings (**a**, **d**) and maximum or average intensity projections (**b**, **f**, **g**) of 90 (**a**, **b**), 84 (**d**), and 10–14 (**f**, **g**) μm thick confocal stacks are shown. In **c**, **e**, **h** and **i**, bars and error bars represent means ± S.E.M. Each circle (*n* = 12 in **c**, 20 in **e**, 10 in **h**, and 9 in **i**), triangle (*n* = 12 in **c**, 20 in **e**, 19 in **h**, and 19 in **i**), and square (*n* = 3) represents a cardiac ventricle. **p ≤ 0.01, ***p ≤ 0.001 (two-tailed student’s *t*-test). Representative images from a total of 12 (**a**), 3 (**b**), 18 (**d**), 11 (**f**), and 19 (**g**) animals are shown. Scale bars: 20 μm (**a**, **d**, **f**, **g**), 50 μm (**b**)

To better characterize fusion-derived cells and understand the functional implications of membrane fusion, we first determined the numbers of cardiomyocytes turning on F/NATC. Following induction of cardiomyocyte-specific CreER activity by 4-OHT treatment from 24 to 40 hpf, expression of LIFEACT-GFP was assessed in 3 dpf, 5 dpf, and 6 months post fertilization (mpf) *Tg(ubb:FATC)* ^*bns97*^ *;Tg(myl7:creER);Tg(myl7:nDsRed2)* fish. On average, 5.2 ± 0.5% of all ventricular cardiomyocytes, identified by nDsRed2 expression, were LIFEACT-GFP^+^ at 3 dpf. This proportion increased to an average of 11.7 ± 1.1% at 5 dpf (Fig. 5a, c). Interestingly, the proportion of LIFEACT-GFP^+^ cardiomyocytes decreased to 5.5 ± 1.7% in adults (Fig. 5b, c). This reduction is in line with the transient nature of the membrane fusion events observed by time-lapse imaging (Supplementary Movie 1; Fig. 3). As FATC reporter expression relies on the presence of both GAL4 protein and *UAS:LIFEACT-GFP* in the same cell, transient fusion that does not generate multinucleated cells would thus lead to only short-lived LIFEACT-GFP expression. Using the same Cre induction regimen, we detected similar numbers of NTR-mCherry^+^ cardiomyocytes in *Tg(ubb:NATC)* ^*bns98*^;*Tg(myl7:creER);Tg(myl7:H2B-GFP)* fish (Fig. 5d, e).

The relatively low percentage of F/NATC-activated cardiomyocytes in the adult myocardium, a tissue with low proliferation rate in physiological conditions (Fig. 5b) prompted us to test for a possible link between membrane fusion and mitotic activity. Thus, we next sought to analyze the mitotic activity of NTR-mCherry^+^ cardiomyocytes in *Tg(ubb:NATC)* ^*bns98*^ animals. Surprisingly, while NTR-mCherry^+^ cardiomyocytes constituted 8.3 ± 0.6% and 16.6 ± 0.8% of the total cardiomyocytes in the 3 and 5 dpf ventricular myocardium (Fig. 5d, e), their contribution to the proliferating pool of cardiomyocytes was markedly higher. In 3 dpf *Tg(ubb:NATC);Tg(myl7:creER)* larvae treated first with 4-OHT to activate Cre at 48 hpf, and then with a 6 h pulse of EdU to label proliferative cells, 16.6 ± 3.4% of all ventricular EdU^+^ cardiomyocytes were also NTR-mCherry^+^ (Fig. 5f, h). In 5 dpf *Tg(ubb:NATC);Tg(myl7:creER)* larvae in which Cre was activated at 24 hpf, this proportion increased to 40.9 ± 3.4% (Fig. 5g, h), corresponding to an ~2.5-fold enrichment of NTR-mCherry^+^ cells in the proliferating cardiomyocyte population. Together, these findings put forth a hypothesis that the cardiomyocytes which experienced membrane fusion represent a highly proliferative subpopulation in the developing heart. Two observations support this hypothesis. First, membrane fusion precedes proliferation. Analysis of NATC larvae treated with 4-OHT prior to exposure to a short pulse of EdU showed that substantial numbers of cardiomyocytes were NTR-mCherry^+^EdU^+^ double-positive (Fig. 5f–h); however, analysis of NATC larvae treated with an EdU pulse given prior to Cre induction detected no NTR-mCherry^+^EdU^+^ double-positive cardiomyocytes (Fig. 2d). Second, F/NATC^+^ cardiomyocytes have higher mitotic rates: analyses of the percentages of NATC^+^ cardiomyocytes (Fig. 5d, e) and the proliferating pool (Fig. 5f–h) determine the proportion of cycling NATC^+^ cardiomyocytes to be approximately 2.4 and 3.4 times higher than NATC^−^ cardiomyocytes at 3 and 5 dpf, respectively. Indeed, we found the percentage of cycling NATC^+^ cardiomyocytes (calculated from EdU^+^NTR-mCherry^+^/NTR-mCherry^+^) to be on average 9.9 ± 1.1%, in comparison to 2.5 ± 0.2% for the general cardiomyocyte population in 5 dpf ventricles (Fig. 5i). Of note, we also assessed *myl7:CreER*-driven recombination efficiency with the same 4-OHT treatment regimens used to assess F/NATC activation. In *Tg(act2b:loxP-DsRed-loxP-EGFP);Tg(myl7:creER)* embryos, GFP^+^ cardiomyocytes covered ~74–75% of the total ventricular area, as measured by the area comprised of both DsRed^+^ cells and GFP^+^ cells (Supplementary Fig. 3A–C). However, the partly overlapping DsRed and GFP signals resulted in higher signal intensity as compared to GFP signal alone. Consequently, image thresholding might have led to an underestimate of the GFP^+^ ventricular area, and consequently recombination efficiency. Therefore, assuming a minimum of 74.1 ± 4.8% recombination efficiency might lead to an underestimation of the actual percentages of F/NATC^+^ and NATC^+^EdU^+^ cardiomyocytes.

### Cardiac regeneration induces F/NATC reporter expression

**Fig. 6:**
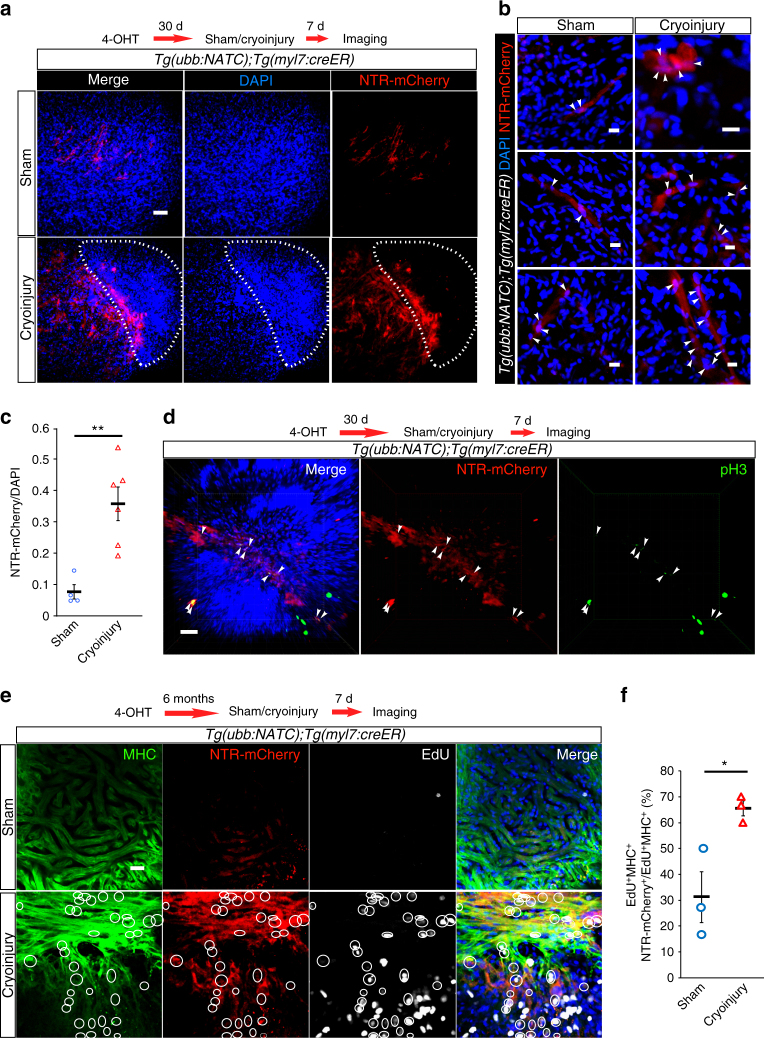
Transgene coupling-mediated fluorescence expression in the adult heart increases after injury. **a**–**c** Cardiac injury induces NATC reporter expression. 6 mpf *Tg(ubb:NATC);Tg(myl7:creER)* fish were injected with 4-OHT intraperitoneally 30 days prior to cryoinjury. **a** Whole-mount immunostaining revealed that NTR-mCherry^+^ cardiomyocytes (red) were localized mainly adjacent to the damaged area (dashed line), identified by disorganized cardiac cells (DAPI^+^, blue). **b** Cytoplasmic NTR-mCherry (red) and nuclear DAPI (blue) labeling shows sham- and injury-induced NATC-activated cardiomyocyte morphologies; arrowheads point to double-labeled cardiomyocytes. **c** Ratios of mean gray values of NATC and DAPI labeling were calculated from maximum intensity projections of 200 μm thick confocal images of individual hearts. **d** Many NTR-mCherry^+^ cardiomyocytes (red) detected mainly at the lesion border zone (disorganized cardiac cells are revealed by DAPI staining, blue) were phospho-histone H3 (pH3, green) positive. **e**, **f** The majority of mitotic cardiomyocytes at the lesion border zone show NATC reporter expression. **e** NTR-mCherry (red) and myosin heavy chain (MHC, green) expression were detected by immunostaining of 6 mpf sham-operated and cryoinjured *Tg(ubb:NATC);* *Tg(myl7:creER)* hearts, in which Cre had been activated by 4-OHT treatment from 24 to 40 hpf. NTR-mCherry^+^ cardiomyocytes undergoing mitosis (white circles) were detected with an 8 h EdU pulse (EdU^+^ nuclei are in white) and their numbers were quantified as percentages of total numbers of mitotic cardiomyocytes (**f**). Cardiac cells were visualized by DAPI (blue) staining. Representative *Tg(ubb:NATC);Tg(myl7:creER)* hearts from four sham and six injured hearts (**a**–**d**), and three sham and three injured hearts (**e**, **f**) are shown/analyzed. Images are maximum or average intensity projections (**a**, **e**) and 3D volume renderings (**d**, **e**) of 200–300 (**a**), 20 (**b**, **e**), and 300 (**d**) μm thick confocal stacks. Bars and error bars in **c**, **f** represent mean ± S.E.M. Each circle (*n* = 4 in **c** and 3 in **f**) and triangle (*n* = 6 in **c** and 3 in **f**) represents a heart. *p ≤ 0.05, **p ≤ 0.01 (two-tailed student’s *t*-test). Scale bars: 5 μm (**b**), 20 μm (**e**), 30 μm (**d**), 50 μm (**a**)

The regenerative response of the adult zebrafish heart is characterized by a burst of cardiomyocyte dedifferentiation and proliferation^10, 11^, providing another model to test the correlation between NATC reporter expression and proliferation in cardiomyocytes. We first induced CreER activity by intraperitoneal (IP) injection of 4-OHT in adult *Tg(ubb:NATC)* ^*bns98*^ *;Tg(myl7:creER)* fish. An earlier study had shown that a single dose of 50 ng of 4-OHT delivered by IP injection to adult *Tg(myl7:creER);Tg(actb2:loxP-DsRed-loxP-EGFP)* zebrafish led to ~20% of the ventricular cardiomyocytes to express EGFP^11^. We chose a 200 times higher dose of 4-OHT for our experiments and thus expected a considerably higher recombination efficiency. Thirty days following cardiomyocyte-specific CreER activation, we performed cardiac cryoinjuries. Seven days after cryoinjury, we detected a noticeable increase of NTR-mCherry expression in the injured hearts (Fig. 6a–c; Supplementary Fig. 6D–H), while sham-operated ones contained only few NTR-mCherry^+^ cells (Fig. 6a–c; Supplementary Fig. 6A–C). Most NTR-mCherry^+^ cells localized at the interface between injured and healthy tissues, where cardiomyocyte proliferation most prominently occurs^10, 11^. Moreover, several NTR-mCherry^+^ cells exhibited atypical morphology, having a more roundish shape compared to the elongated appearance of the few NTR-mCherry^+^ cardiomyocytes detected within the healthy tissue (Fig. 6b). This observation is consistent with the round cells corresponding to dedifferentiated cardiomyocytes, characterized by sarcomeric disassembly and cell detachment prior to proliferation^11^. Injury-induced activation of NATC, localization of NATC-activated cells around the lesion area, and atypical morphology of these cells were also observed in cryoinjured *Tg(ubb:NATC)* ^*bns98*^ *;Tg(hsp:cre)* fish (Supplementary Fig. 7G–O). Consistent with our hypothesis of a link between F/NATC reporter expression and mitotic activity in cardiomyocytes, we observed that around the injured area, a substantial number of proliferating cells, detected by the M-phase marker Phospho-Histone H3 (pH3), showed NTR-mCherry (Fig. 6d) or LIFEACT-GFP (Supplementary Fig. 7A–F) expression. These data suggest that the correlation between cardiomyocyte membrane fusion and proliferation in larval hearts is also present in the regenerating adult myocardium.

Next, we examined the contribution of NATC-activated cells to the proliferating subset of cardiomyocytes during heart regeneration. We used 6 mpf *Tg(ubb:NATC);Tg(myl7:creER)* fish treated with 4-OHT to induce Cre activity from 24 to 40 hpf (Fig. 6e, f). This 4-OHT treatment regimen is the same as the one used to generate the data shown in Fig. 5a, d, g, and so is expected to yield at least a 74% recombination efficiency (Supplementary Fig. 3). Cells that underwent DNA synthesis were labeled by an 8 h pulse of EdU at 7 days post injury. Cardiomyocytes were identified by the presence of sarcomeric arrays revealed by immunostaining for myosin II heavy chain (MHC). Consistent with our initial observations with pH3, NATC-activated cardiomyocytes (NTR-mCherry^+^MHC^+^) most prominently localized at the interface between healthy and injured tissues, and constituted on average 71.5 ± 2.4% of the cycling cardiomyocytes in this area as indicated by the ratio of EdU^+^NTR-mCherry^+^MHC^+^/EdU^+^MHC^+^ (Fig. 6e, f).

Our findings of a high proliferative rate of F/NATC-activated cardiomyocytes in larval hearts (Fig. 5d–h), of the extensive contribution of F/NATC^+^ cells to the cycling cardiomyocytes in regenerating adult hearts (Fig. 6d–f; Supplementary Fig. 7A–F), and of the detection of NATC reporter expression in larval cardiomyocytes prior to their proliferation (Figs. 2c and 5f–h) collectively suggest an increase in cardiomyocyte proliferation rate after membrane fusion during both development and tissue repair.

### *jam3b* mutation impairs cardiac development and function

Our findings thus far support the hypothesis that membrane fusion primes cardiomyocytes for proliferation. However, another scenario in which cell cycle entry confers fusion capability to cardiomyocytes is also plausible. To try and distinguish between these possibilities, we examined zebrafish harboring a missense mutation in the cell surface receptor gene *junction adhesion molecule* (*jam*) *3b*, reported to be indispensable for skeletal muscle fusion^31^. Expression of *jam3b* in cardiomyocytes was confirmed by in situ hybridization of 3 dpf *Tg(myl7:H2B-GFP)* larvae (Supplementary Fig. 8A). The *jam3b* mutant is currently one of the very few tools available to elucidate the functional implication of cell fusion in zebrafish. Other molecules known to affect cell fusion, many of which act within pathways modulating actin filament assembly, regulate multiple aspects of tissue development and function. On the other hand, homozygous mutations in *jam3b* cause fast twitch muscle fusion defects, without detectable effects on myocyte differentiation, myofilament structure, or overall development^31^. Mammalian Jam3b (JAM-C) has been reported to localize to tight junctions in epithelial cells where it interacts with the tight junction-associated protein ZO-1^32^. First, to test whether tight junction formation was affected by Jam3b deficiency, we assessed the amount of ZO-1 localized to the plasma membrane of cardiomyocytes in *jam3b* ^*−/−*^ larvae. As shown in Supplementary Fig. 8B, C, *jam3b* ^*−/−*^ animals showed comparable ZO-1 levels as their wild-type siblings. ZO-1 expression and localization in other tissues were also similar in mutant and control animals (Supplementary Fig. 8D). Accordingly, the *jam3b* mutation does not appear to affect cardiomyocyte morphology (Supplementary Fig. 8E), as assessed by measuring cardiomyocyte volume and surface area (Supplementary Fig. 8F, G). Thus, our data confirm the suitability of the *jam3b* mutant as a fusion-deficient model.

**Fig. 7:**
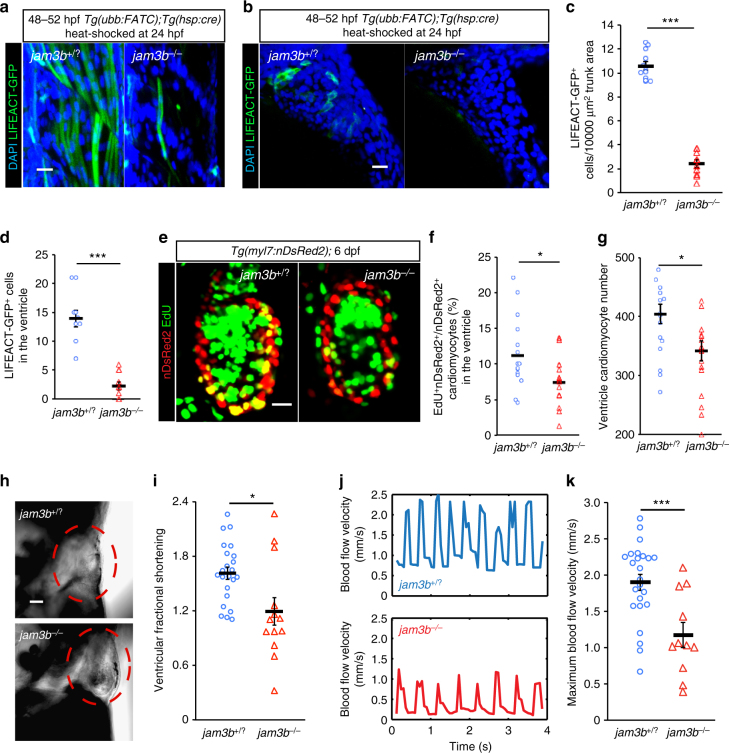
Cell fusion deficiency negatively affects cardiomyocyte proliferation and cardiac function. **a**–**d** *jam3b* mutants show a reduction in the number of LIFEACT-GFP^+^ skeletal and cardiac myocytes. Embryos from a cross of *jam3b* ^*+/−*^ *;Tg(ubb:FATC)* and *jam3b* ^*+−*^ *;Tg(hsp:cre)* fish were heat-shocked at 24 hpf. LIFEACT-GFP^+^ (green) skeletal (**a**) and cardiac (**b**) muscles were detectable at 48–52 hpf. DAPI-stained nuclei are shown in blue. **c**, **d** LIFEACT-GFP^+^ skeletal myocytes per 10,000 μm^2^ of trunk surface area (**c**) and LIFEACT-GFP^+^ ventricular cardiomyocytes (**d**) in *jam3b* ^*−/−*^ embryos and *jam3b* ^*+/?*^ siblings. **e**–**g** Jam3b deficiency reduces cardiomyocyte proliferation. A 16 h EdU pulse (green) labeled mitotic cardiomyocytes (nDsRed^+^, red) (**e**) shown as percentage of total cardiomyocytes (**f**); total cardiomyocyte numbers (**g**) in ventricles of 6 dpf *jam3b* ^*−/−*^ *;Tg(myl7:nDsRed2)* and *jam3b* ^*+/?*^ *;Tg(myl7:nDsRed2)* siblings. **h**–**j** *jam3b* deficiency impairs cardiac function. **h** The 5 dpf *jam3b* mutants exhibit pericardial edema (28 out of 36 fish with edema were jam3b^−/−^). **i**, **j** The 5 dpf *jam3b* mutants also display decreased fractional shortening (**i**) and blood flow velocity (**j**) compared to control siblings, as evidenced by a significant difference in average maximum flow velocity (**k**). **a**, **b**, **e** are maximum or average intensity projections of 20–65 μm thick confocal stacks. **h** are single focal planes of confocal images showing larvae in lateral views, anterior up and dorsal to the left. In all plots, bars and error bars represent means ± S.E.M. Each circle and triangle represents an embryo (**c**) or a heart (**d**, **f**, **g**, **k**, **j**). *p ≤ 0.05, **p ≤ 0.01, ***p ≤ 0.001 (two-tailed student’s *t*-test); 10–12 (**a**–**d**), 16 (**e**–**g**), 36 (**h**), and 11–25 (**i**–**k**) −/− and +/? sibling animals were examined. Scale bars: 20 μm (**a**, **b**, **e**), 40 μm (**h**)

At 48–52 hpf, while *Tg(ubb:FATC)* ^*bns97*^ *;Tg*(*hsp:cre);jam3b* ^*+/?*^ (wild-type or heterozygous for *jam3b*) embryos exhibited exclusively multinucleated fast twitch muscles, the majority of myocytes in *jam3b* ^*−/−*^ embryos were mononucleated (Fig. 7a; Supplementary Fig. 9A−P), indicating cell fusion defects. Correspondingly, Jam3b deficiency caused a 78% reduction in the number of LIFEACT-GFP^+^ skeletal myocytes in 48–52 hpf embryos after heat-induction at 24 hpf to drive ubiquitous *cre* expression (Fig. 7a, c; Supplementary Fig. 9A–P). Importantly, LIFEACT-GFP expression could only be observed in the few multinucleated myocytes, providing additional verification for the suitability of the tool to report cell fusion (Supplementary Fig. 9Q). We observed a similar decrease (77%) in the number of LIFEACT-GFP^+^ cardiomyocytes in *jam3b* mutants (Fig. 7b, d), further supporting the occurrence of cell/membrane fusion in the myocardium. Analysis after a 16 h EdU pulse showed that the percentage of ventricular cardiomyocytes undergoing DNA synthesis in 6 dpf *jam3b* ^*−/−*^ larvae was ~33% lower than in wild-type siblings (Fig. 7e, f). Accordingly, 6 dpf mutant ventricles contained, on average, 16% less cardiomyocytes than wild-type siblings (Fig. 7g). Moreover, most *jam3b* ^*−/−*^ mutants exhibited pericardial edema by 5 dpf (Fig. 7h). While heart rate was only slightly affected (132 ± 2.4 versus 120 ± 3 beats per minute in wild-type siblings and *jam3b* mutants, respectively, *p* = 0.01 *t*-test), loss of Jam3b markedly decreased ventricular fractional shortening (Fig. 7i) and blood flow velocity (Fig. 7j, k) when analyzed at 5 dpf, indicating defective cardiac contractility. Altogether, these findings suggest the requirement of membrane fusion for cardiomyocyte proliferation and cardiac function.

### Unaltered cardiomyocyte ploidy following membrane fusion

**Fig. 8:**
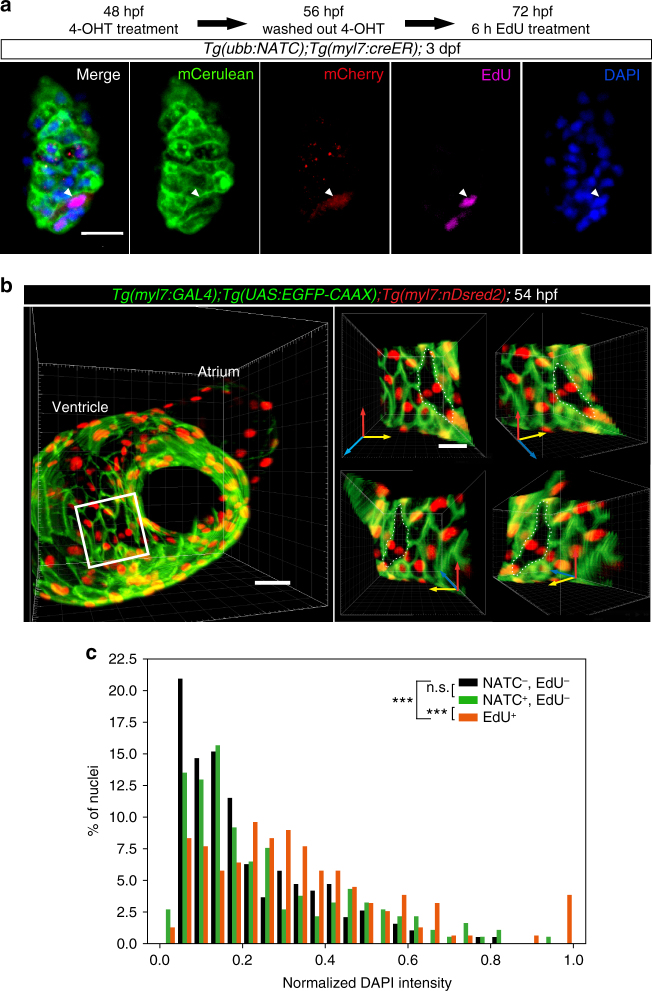
Membrane fusion does not generate multinuclear or polyploid cardiomyocytes. **a** Mononucleated NATC-activated cardiomyocytes undergo mitosis. *Tg(ubb:NATC);Tg(myl7:creER)* embryos were treated with 4-OHT from 48 to 56 hpf. At 72 hpf, the larvae were given a 6 h EdU pulse. Membrane Cerulean (green) and cytoplasmic mCherry (red) expression were visualized by immunostaining. EdU (magenta) and DAPI (blue) staining shows a single nucleus in the EdU + mCherry^+^ cardiomyocyte (arrowhead). 7.2 μm thick confocal stacks are shown as maximum intensity projections. **b** Binucleation is rarely observed in embryonic cardiomyocytes. Images are 3D volume renderings of a 54 hpf heart. Cardiomyocyte membrane and nuclei were visualized by *Tg(myl7:GAL4);Tg(UAS:*EGFP-CAAX*)* (green) and *Tg(myl7:*nDsRed2*)* (red) expression, respectively. *Y* axis rotation of the ventricular area (small panels on the right), indicated by the white box shows the single-layered compact myocardium with a binuclear cardiomyocyte, outlined with white dashed lines. **c** Among the EdU^−^ populations, the distributions of DAPI intensity in NTR-mCherry^+^ cardiomyocytes (green bars) and NTR-mCherry^−^ cardiac cells (black bars) were similar, indicating that DNA content in fused cardiomyocytes is not different from that of non-fused cardiac cells. On the contrary, and as expected, DNA content in EdU^+^ cells (red bars) was significantly higher than that in EdU^−^ cells (either NTR-mCherry^+^ or NTR-mCherry^−^). *Tg(ubb:NATC);Tg(myl7:creER)* embryos were treated with 4-OHT from 24 to 40 hpf. NTR-mCherry expression and proliferating cells were labeled by immunostaining at 7 dpf after a 16 h EdU pulse. DAPI intensity measurements were performed on 3D volume-renderings obtained from confocal images. *n* = 311 NTR-mCherry^−^EdU^−^ cells, *n* = 227 NTR-mCherry^+^EdU^−^ cells, *n* = 173 EdU^+^ cells, from 5 larvae. ***p < 0.001, n.s., not significant, two-sample Kolmogorov–Smirnov test. Representative images from 11 larvae (**a**) and 10 embryos (**b**) are shown as average intensity projections (**a**) and 3D surface renderings (**b**) of 12 (**a**) and 60 (**b**) μm thick confocal stacks. Scale bars: 20 μm (**a**, **b** small panels), 30 μm (**b**, large panel on the left)

Multi-nucleation, commonly observed in mammalian cardiomyocytes and believed to be the result of abortive cytokinesis^26^ instead of cell fusion, has been linked to lower cell cycle activity^33, 34^. In contrast, we found evidence suggesting high mitotic activity of cardiomyocytes after membrane fusion (Figs. 2c, 4d–h, and 6e, f). Moreover, live imaging revealed transient fusion events that did not lead to multi-nucleation (Fig. 2e; Supplementary Movie 1). Further examination of proliferation events in fusion-derived cardiomyocytes, detected in *Tg(ubb:NATC)* ^*bns98*^ *;Tg(myl7:creER)* larvae treated with 4-OHT at 48–56 hpf and a subsequent EdU-pulse at 72–78 hpf, showed EdU incorporation in mononucleated cells (Fig. 8a). To clarify whether the developing zebrafish heart contains multinucleated cardiomyocytes, we analyzed *Tg(myl7:GAL4);Tg(UAS:EGFP-CAAX);Tg(myl7:nDsRed2)* embryos at 54 hpf, a developmental stage in which the myocardium is monolayered and individual cardiomyocytes are identifiable by the presence of membrane-localized EGFP-CAAX and nuclear nDsRed2. Excluding cell pairs showing partial membrane fusion, we observed negligible numbers of binucleated cardiomyocytes (a total of 3 cardiomyocytes in 2 out of 10 embryos analyzed; a representative image is shown in Fig. 8b). Consistent with our analysis, it has been reported that the majority of cardiomyocytes in the adult zebrafish heart are mononucleated^35^. To test the possible occurrence of nuclear fusion, which would lead to mononuclear polyploid cells, we assessed the nuclear DNA content of cardiomyocytes in *Tg(ubb:NATC);Tg(myl7:creER)* larvae at 7 dpf. Fused cells were identified by NTR-mCherry expression following 4-OHT treatment from 24 to 40 hpf. Intensity of DAPI staining was used to quantify DNA amounts. Cells undergoing DNA synthesis, which would alter DNA content, were labeled by a 16 h EdU treatment. Fluorescence image analysis showed no difference in the nuclear DNA content of NATC^+^ and NATC^−^ cardiomyocytes, suggesting that ploidy number was unchanged in cardiomyocytes after membrane fusion (Fig. 8c). These data argue against cell fusion leading to multinucleated or polyploid cardiomyocytes, and further suggest the presence of transient membrane fusion events in the zebrafish heart.

## Discussion

We describe here novel tools, *Tg(ubb:FATC)* ^*bns97*^ and *Tg(ubb:NATC)* ^*bns98*^, that enable in vivo labeling and ablation of fusion-derived cells. In addition to membrane or cell fusion, LIFEACT-GFP expression observed in some skin cells (Supplementary Fig. 1A), which have been reported to become polyploid through endoreplication^23, 24^, suggests that endoreplication might also be able to activate the FATC and NATC reporters. Use of these tools led us to identify the myocardium, a tissue previously unknown to undergo frequent membrane fusion.

Cell fusion is a well-known cellular event that involves complete regression of the intercellular membranes to generate multinucleated, or mononucleated polyploid, cells. In contrast, we found several lines of evidence that suggest an uncommon form of fusion that does not give rise to multinucleated/polyploid cardiomyocytes. Taking into account (1) the rarity of binucleated cardiomyocytes (Fig. 8b), especially compared to the high numbers of mostly mononucleated (Fig. 5a, c, d), FATC^+^ (Fig. 5a, c) and NATC^+^ (Fig. 5d, e) cardiomyocytes, (2) the transient nature of membrane fusion events that do not give rise to stable binucleated cells as documented by time-lapse imaging (Supplementary Movie 1; Fig. 2e), and (3) the unaltered ploidy of cardiomyocytes after membrane fusion as assessed by DNA content analysis (Fig. 8c), we conclude that transient membrane fusion events that allow cytoplasmic connections is the most likely mechanism by which cardiomyocytes activate the F/NATC reporter.

Heterokaryon formation between transplanted endothelial/hematopoietic lineage cells^13, 14^ or skeletal muscle-derived cells^36^ and host cardiomyocytes has been reported previously. Hematopoietic stem/progenitor cells, in particular, are able to fuse homotypically^37^ and heterotypically^8^ with diverse cell types. However, constitutive *cre* expression under control of the *kdrl* promoter, which is active in endothelial and hematopoietic stem cells^38^, did not induce LIFEACT-GFP expression in cardiomyocytes (Supplementary Fig. 10). Instead, time-lapse imaging showed membrane fusion between cardiomyocytes (Fig. 2e; Supplementary Movie 1), indicating homotypic fusion.

Establishment of nanotubular structures that allow transient exchange of cytoplasmic macromolecules and organelles between cardiomyocytes and other cell types including mesenchymal stem cells, endothelial progenitor cells, and fibroblasts has been observed in vitro^39, 40^ and in vivo^19^, indicating functional significance of partial membrane fusion. Accordingly, our work has uncovered a correlation between membrane fusion and mitotic activity of cardiomyocytes in response to the proliferative demand during development (Fig. 5d–h) and regeneration (Fig. 6d–f). It will be interesting to clarify the nature of this relationship and its underlying mechanism. While supporting data are needed, two possible scenarios might occur upon membrane fusion. One scenario is the presence of a proliferation competent cardiomyocyte population for which membrane fusion and exchange of cytoplasmic materials drives their entry into the cell cycle. Another scenario involves a fusion-triggered metabolic switch to aerobic glycolysis, which has long been considered a requirement for cell proliferation^41^. This speculation is in line with an earlier posited hypothesis that fusion between cancer cells and leukocytes can generate hybrid cells that use aerobic glycolysis to fuel their transition to malignancy^42^.

We describe here cardiac defects in *jam3b* mutants. The reduction in *jam3b* mutants of FATC reporter expression (Fig. 7a–d), of cardiomyocyte S-phase indexes, of total cardiomyocyte numbers, coupled with defective cardiac contractility (Fig. 7e–k), but without obvious changes in cardiomyocyte adhesion or morphology (Supplementary Fig. 8) suggests a requirement for Jam3b-mediated cardiomyocyte fusion in cardiac development and function. It has been postulated that fusion serves an essential function in skeletal myogenesis by allowing the generation of continuous sarcomeric arrays important for contractility^1^. Sarcomere alignment between adjacent cardiomyocytes to enhance synchronous contractions^43^ might be facilitated by cardiomyocyte fusion, even though the transient fusion events do not generate long-lasting multinuclear syncytia. Alternatively, the reduction in the number of ventricular cardiomyocytes in *jam3b* mutants (Fig. 7e–g) could directly contribute to cardiac dysfunction, and further analysis will determine whether these or additional mechanisms are at play in mediating the developmental and functional roles of Jam3b in the heart. The human homolog of *jam3b*, *JAM-3*, was identified as a candidate gene within a chromosomal region associated with congenital heart defects in Jacobsen Syndrome^44^. Mice lacking *JAM-C*, a *jam3b* ortholog, exhibit no gross cardiac abnormalities^44^ but die perinatally from respiratory distress^45^. The phenotypes we observed in the zebrafish *jam3b* mutant hearts lead us to speculate that cardiomyocyte fusion is an indispensable process in vertebrates. Furthermore, the correlation between cardiomyocyte fusion and mitotic activity calls for additional studies of the underlying mechanisms of cell fusion, which may lead to a better understanding of the distinct regenerative capacity of the zebrafish heart and provide new avenues for repairing the damaged mammalian heart.

## Methods

### Fish care and strains

All zebrafish husbandry was performed under standard conditions in accordance with institutional (MPG) and national German ethical and animal welfare guidelines. Transgenic lines used in this study include: *Tg(ubb:lox2272-mCerulean-UAS-loxP-lox2272-GAL4-loxP-LIFEACT-GFP)* ^*bns97*^, *Tg(ubb:lox2272-mCerulean-UAS-loxP-lox2272-GAL4-loxP-NTR-mCherry)* ^*bns98*^, *Tg(hsp70l:cre)* ^*zdf13*,^ ^46^, *Tg(myl7:Cre-ERT2)* ^*pd12*,^ ^11^, *Tg(myl7:DsRed2-NLS)* ^*f2*,^ ^47^, *Tg(myl7:MKATE-CAAX)* ^*sd11*,^ ^48^, *Tg(myl7:EGFP)* ^*twu26*,^ ^49^, *Tg(myl7:H2B-GFP)* ^*zf52*,^ ^50^, *Tg(kdrl:Cre)* ^*s898*,^ ^51^, *Tg(myl7:GAL4)*, *Tg(UAS:EGFP-CAAX)* ^*m1230*,^ ^52^, and *Tg(actb2:loxP-DsRed-loxP-EGFP)* ^*s928*,^ ^11^.

### Generation of transgenic animals

To generate the ubb:lox2272-mCerulean-UAS-loxP-lox2272-GAL4-loxP-LIFEACT-GFP construct, the ubb promoter from Tg(ubb:loxP-EGFP-loxP-mCherry)^cz1701,^ ^53^, lox2272-mCerulean from CMV-Brainbow-1.1 M, loxP-5XUAS-E1b, rabbit β-globin intron from pT2KXIGΔin ref. ^54^, lox2272-GAL4TA4-loxP, and LIFEACT-GFP from Tg(myl7:LIFEACT-GFP)^s974,^ ^55^ were amplified by polymerase chain reaction (PCR) and cloned into a miniTol2 vector. The ubb:lox2272-mCerulean-UAS-loxP-lox2272-GAL4-loxP-NTR-mCherry construct was generated by replacing LIFEACT-GFP in the ubb:lox2272-mCerulean-UAS-loxP-lox2272-GAL4-loxP-LIFEACT-GFP plasmid with NTR-mCherry, PCR-amplified from Tg(UAS-E1b:NTR-mCherry)^i149,^ ^56^.

Injected fish were selected for mCerulean expression and raised to adulthood. Identified founders were outcrossed and subsequent generations were raised and expanded. Most of the animals used in this study were F4 animals obtained from consecutive outcrosses.

### Heat shock and tamoxifen treatment

For heat induction of *cre* expression, embryos or adults were transferred into fresh Danieau buffer or system water preheated to 38–39 ºC and kept at this temperature for 1 h. Activation of Cre-ERT2 in embryos was done by addition of ethanol-stocked 4-hydroxytamoxifen (4-OHT, Sigma) in Danieau buffer to a final concentration of 5 μM. For adults, 0.01 mg of 4-OHT (~0.05 mg/g of body weight) was delivered by intraperitoneal injections.

### EdU treatment and immunofluorescent staining

To assess mitotic activity, EdU (Thermo Fisher) was diluted in Danieau buffer containing phenylthiourea (PTU) to a concentration of 5 mM. Embryos/larvae were immersed in EdU medium, containing 1.5% DMSO to enhance permeability, and incubated at 28 ºC for the indicated times before fixation. Adult fish were injected with 0.04 mg of EdU intraperitoneally (~0.2 mg/g of body weight).

For EdU and immunofluorescence staining, samples were fixed with 4% paraformaldehyde (PFA) at 4 °C overnight, washed with PBS buffer containing 0.1% (V/V) Tween 20 (PBST), and incubated with 1 mg/ml collagenase (Sigma) in PBST at 37 °C for 1.5 h to improve antibody penetration. Before proceeding to staining, the pericardium was removed from embryos/larvae and adult hearts were treated with 10 μg/ml proteinase K in PBST for 30 min at room temperature (RT). EdU staining was performed according to the Click-iT protocol provided by the manufacturer (Thermo Fisher Scientific). Next, samples were incubated at RT for 2 h in blocking buffer (PBS supplemented with 0.3% (V/V) Triton X-100 and 1% DMSO (PBSTD) and contained 2% (W/V) BSA and 4% (V/V) goat serum), followed by primary antibodies diluted in blocking buffer for 48–72 h at 4 °C. After washing out unbound primary antibodies with PBSTD, samples were incubated with Alexa Fluor-conjugated secondary antibodies (Thermo Fisher Scientific) in blocking solution (dilution 1:500) for 2 h at RT, washed with PBSTD and mounted in Fluoromount aqueous mounting medium (Sigma) for imaging. Antibodies and dilutions are: 1:300 rabbit anti-phospho-Histone H3 (Millipore), 1:50 mouse anti-phospho-histone H3 (C-2, Santa Cruz), 1:500 rabbit anti-RFP (MBL), 1:500 chick anti-GFP (Molecular Probes), and 1:50 mouse anti-ZO-1 (Thermo Fisher Scientific). The monoclonal mouse anti-F59 and anti-EB165 antibodies were obtained from the Developmental Studies Hybridoma Bank, created by NICHD of the NIH and maintained at the University of Iowa, Iowa, USA.

To quantify numbers of NATC^+^ and proliferating cardiomyocytes, all single-plane images from confocal stacks that covered the entire cardiac ventricle were analyzed. Cells showing NTR-mCherry expression or EdU-labeled nuclei that were entirely embedded within NTR-mCherry^+^ cytoplasm were scored in ImageJ. Total cardiomyocyte numbers were quantitated using Imaris software.

### Time-lapse microscopy

Embryos were embedded in 1.5% low-melting agarose in glass-bottom dishes (MatTek). The dishes were then filled with Danieau buffer containing 0.016% Tricaine to anesthetize the embryos. Confocal Z-stacks were acquired from the heart every 20–30 min for 17 h using a ×40 water immersion objective. Maximum intensity projections of each time frame were rendered into time-lapse movies with Fiji software.

### Morpholino injections and blastula transplantations

For time-lapse imaging, 5 ng of *tnnt2* morpholino^30^ and 20–40 pg of *cre* or *creER* mRNA was injected in embryos at 1–4-cell stage. For transplantation, embryos were dechorionated by treatment with 1 mg/ml pronase (Sigma) in Danieau buffer and maintained on agarose-coated Petri dishes until 1k-cell stage. Embryos were then transferred into agar mold that contained rows of wells large enough to hold 1–2 embryos. In Ringer’s buffer containing Penicillin/Streptomycin, donor cells were transplanted into host embryos along the blastoderm margin. Transplantations were continued until embryos developed to the 30%-epiboly stage. Transplanted embryos were raised to 24 hpf in Ringer’s buffer containing Penicillin/Streptomycin on agarose-coated petri dishes and transferred into uncoated dishes with Danieau buffer for further development.

### Cardiac injury

Fish were anesthetized with tricaine and placed on a soft sponge soaked in system water containing tricaine with their ventral side exposed. After removing scales from the chest area, a small longitudinal incision was made from the operculum to the pectoral fin level. A liquid nitrogen-cooled stainless steel probe was placed on the ventricle for 20–30 s to induce cryoinjury. Injured fish were left in small tanks containing system water to recover from anesthesia before returning them back to water-flowing tanks.

### Cardiac contractility measurements

Five days post fertilization, larvae obtained from *jamb* ^*+/−*^ incrosses were embedded in 2% low-melting point agarose. PTU was applied to the animals starting from 1 dpf to block skin pigmentation. The heart ventricle and a segment of the dorsal aorta were imaged with a transmitted light microscope equipped with a ×20 objective (Olympus) and a high-speed camera (Andor). Four seconds long movies were acquired at 420 frames per second.

To calculate ventricular fractional shortening, the width of the ventricle at the maximum ventricular diastole (width_VD_) and ventricular systole (width_VS_) were measured in Fiji. The ventricular fractional shortening was calculated as [(width_VD_ – width_VS_)/width_VD_].

Blood velocity was calculated in two steps. First, kymograms (space-time images representing the motion of particles as oblique lines) of blood cell movement in the dorsal aorta were generated with Fiji using the Velocity Measurement Tool (http://dev.mri.cnrs.fr/projects/imagej-macros/wiki/Velocity_Measurement_Tool). Second, the kymograms were filtered with a temporal demeaning algorithm, and blood flow velocity was calculated from the angles of the lines in the kymograms using a Matlab code^57^.

### Quantification of DNA content

Twenty-four hours post fertilization, *Tg(ubb:NATC);Tg(myl7:creER)* embryos were treated with 4-OHT for 16 h. After a 16 h pulse of EdU at 6 dpf, NTR-mCherry^+^ and EdU^+^ cells were detected by immunofluorescence staining. DNA was stained with DAPI (1 μg/ml for 2 h at RT). Nuclear DAPI intensities of cardiac cells were quantified from 3D volumes obtained from confocal images using ImarisCell software.

### Data availability

All data supporting the findings of this study are available within the article and its supplementary information files or from the corresponding authors upon reasonable request.

## Electronic supplementary material

Supplementary Information

Description of Additional Supplementary Files

Supplementary Movie 1
