# Supplementary material for: Transient cardiomyocyte fusion regulates cardiac development in zebrafish
Source: Nat Commun. 2017 Nov 15;8:1525. doi: 10.1038/s41467-017-01555-8 (PMC5688123; doi:10.1038/s41467-017-01555-8)
Supplement: Supplementary file 1 — Supplementary Information [file 41467_2017_1555_MOESM1_ESM.pdf]

Supplementary information

A

Cre expression method	Induction time	Tissues with abundant LIFEACT-GFP+ cells	Tissues with sparse LIFEACT-GFP+ cells
hsp70:cre	24 hpf	skeletal muscle<br>myocardium<br>skin	liver (≤10 cells/animal)<br>CNS (≤10 cells/animal)
cre mRNA		skeletal muscle<br>myocardium<br>skin	liver (≤10 cells/animal)<br>CNS (≤10 cells/animal)<br>endocardium<br>(≤10 cells/animal)<br>epicardium<br>(≤3 cells/animal)<br>blood/leukocyte<br>(≤10 cells/animal)
myl7:creER	24 hpf	myocardium	not detected
kdrl:cre		not detected	not detected

Tg(ubb:FATC);Tg(hsp:cre) heat-shocked at 24 hpf; 5-7 dpf

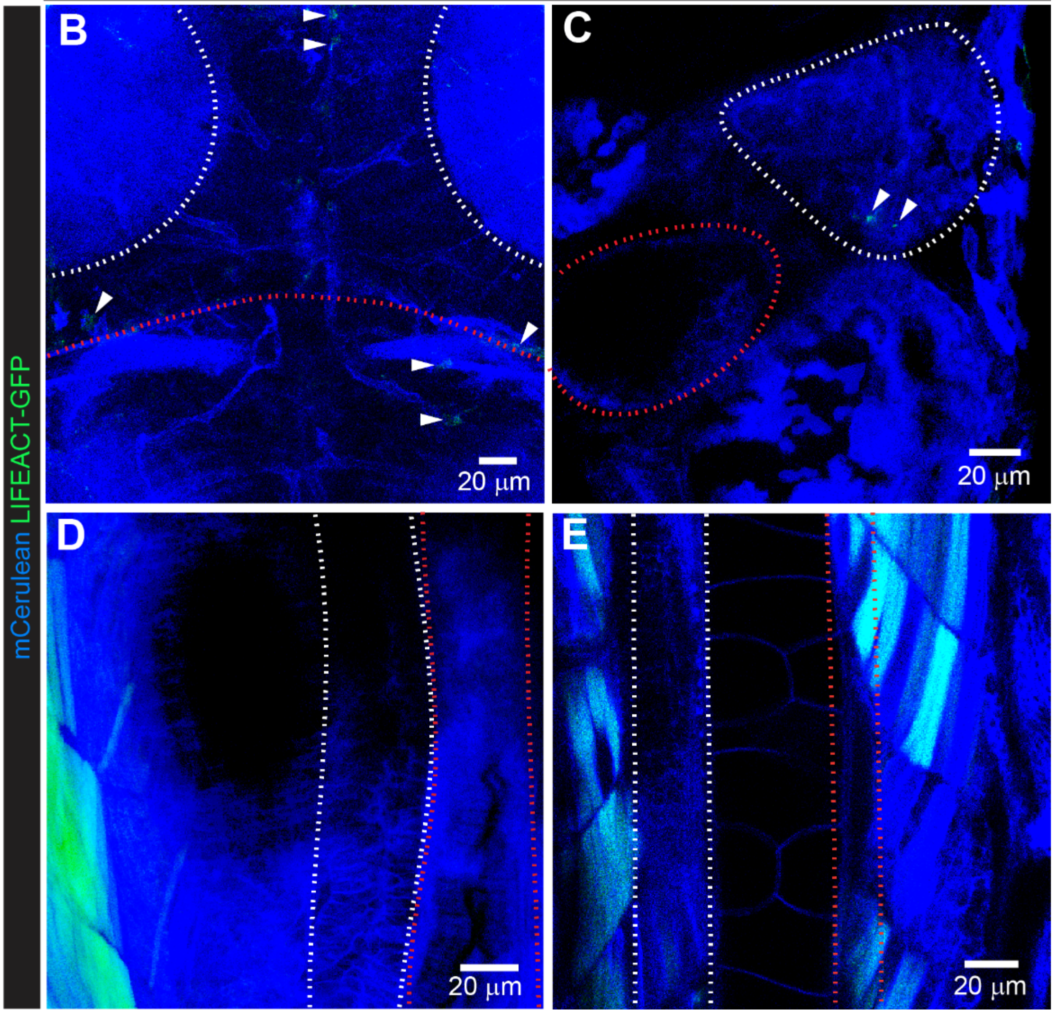

Supplementary Figure 1. FATC reporter expression is restricted to a few tissues. A, List of tissues containing LIFEACT-GFP⁺ cells, observed between 4-7 dpf, upon ubiquitous and cell-type

specific *cre* expression in *Tg(ubb:FATC)* animals. Ubiquitous *cre* expression by the heat-inducible *Tg*(*hsp70:cre)* or injection of *cre* mRNA in *Tg(ubb:FATC)* embryos gave rise to LIFEACT-GFP$^+$ skeletal and cardiac myocytes, and skin cells. A few cells in the liver and central nervous system (CNS), were also LIFEACT-GFP$^+$. 4-OHT induction of cardiomyocyte-specific *cre* expression in *Tg(ubb:FATC);Tg(myl7:creER)* embryos at 24 hpf led to LIFEACT-GFP$^+$ cardiomyocytes. No LIFEACT-GFP$^+$ cells were detectable in *Tg(ubb:FATC);Tg(kdrl:cre)* animals in which *cre* is constitutively expressed in endothelial lineage cells. **B**-**E**, Embryos from *Tg(ubb:FATC)* and *Tg(hsp:cre)* crosses were heat-shocked at 24 hpf to induce ubiquitous *cre* expression. While the expression of the *ubb:FATC* transgene was observed by membrane Cerulean fluorescence (blue) in all tissues, a few LIFEACT-GFP$^+$ cells (green, arrowheads) were detectable in the CNS (**B**) and the liver (**C**) at 5-7 dpf. The muscle (**D** and **E**) and the skin (not shown) were the only tissues that contained significant numbers of LIFEACT-GFP$^+$ cells. Areas enclosed by white dashed lines are the tectal neuropil (**B**), liver (**C**), pancreas (**D**), and spinal cord (**E**). Red dashed lines mark the midbrain-hindbrain boundary (**B**), gall bladder (**C**), intestine (**D**), and dorsal aorta (**E**). All images are maximum intensity projections of 20-60 µm thick confocal stacks. **B** is dorsal view, anterior up. **C**-**E** are lateral views, dorsal to the left, anterior up. 12-39 animals were examined for each *cre* expression method.

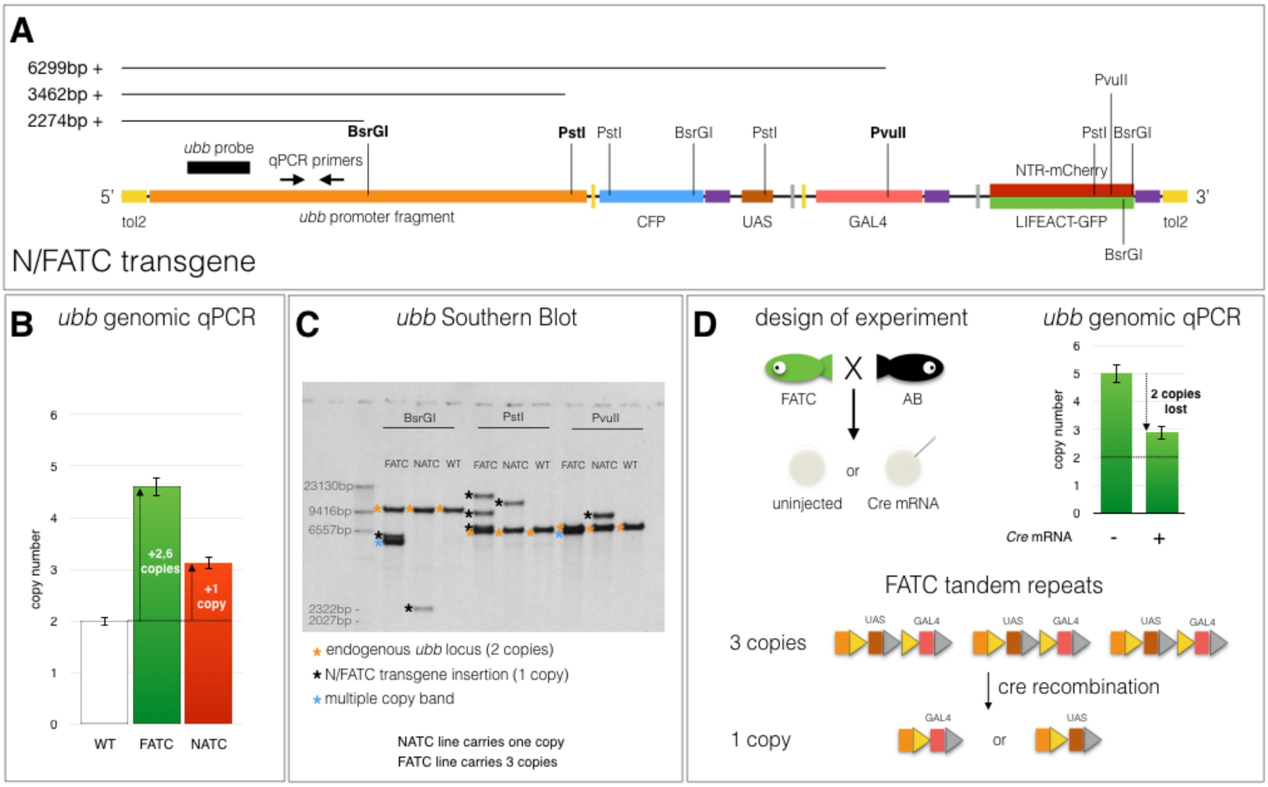

Supplementary Figure 2. Assessment of *Tg(ubb:FATC)* and *Tg(ubb:NATC)* copy numbers.

A, Schematic drawing of the *N/FATC* transgenes. The two constructs differ only in the *NTR-mCherry/LIFEACT-GFP* cassette. The position of the probe and qPCR primers for the *ubb* promoter is shown. Restriction sites for the enzymes used in the Southern Blot analysis that are closest to the transgene start (5' tol2 site) are in bold. Minimum fragment length is marked for each digest. **B**, Quantitative PCR on genomic DNA reveals that the FATC and NATC lines bear 4.6±0.18 and 3.12±0.12 *ubb* promoter copies, respectively. Since the endogenous *ubb* locus contributes 2 copies in the WT sample, these data indicate 2-3 transgene copies for *FATC* and a single copy for *NATC*. Two primer sets for chromosomes 8 and 11 were used for genomic DNA input normalization. **C**, Southern Blot analysis for *FATC* and *NATC* transgene copy number. All three different restriction enzyme treatments suggest a single copy in the NATC fish line. In contrast, the FATC line shows 3 copies. Black stars mark a single copy, blue stars multiple copies (higher intensity bands), and orange stars endogenous *ubb* promoter copies (note the higher intensity due to two alleles) at the expected sizes. **D**, Resolution of the *FATC* multiple copy number into one *FATC* product (GAL4 driver or UAS reporter) after Cre recombination; design of the experiment, quantitative PCR and example of resolution after Cre recombination. Embryos from a FATC x AB (WT) outcross were injected with *cre* mRNA or not injected (control) at the one cell stage. For both groups, embryos were selected for Cerulean expression (i.e., carrying the FATC transgene) and used for quantitative PCR. The qPCR data indicate that Cre injected embryos carry 2 fewer copies of the *ubb* promoter than their uninjected siblings (2.9±0.25 versus 5±0.35 copies, respectively). When in tandem orientation, three (or any number of) *FATC* transgenes recombine into a single *FATC* product, either a *ubb* driven *GAL4* expression cassette or a *UAS* driven LIFEACT-GFP reporter.

Pools of 20-30 *Tg(ubb:FATC)* or *Tg(ubb:NATC)* larvae were used to extract gDNA. Siblings from the *Tg(ubb:FATC)* outcross, negative for Cerulean expression served as a negative control (WT). Error bars represent SEM for technical triplicates. Expression levels for *ubb* in WTs were set as 2 copies (**B**) and in uninjected FATC as 5 copies (**D**).

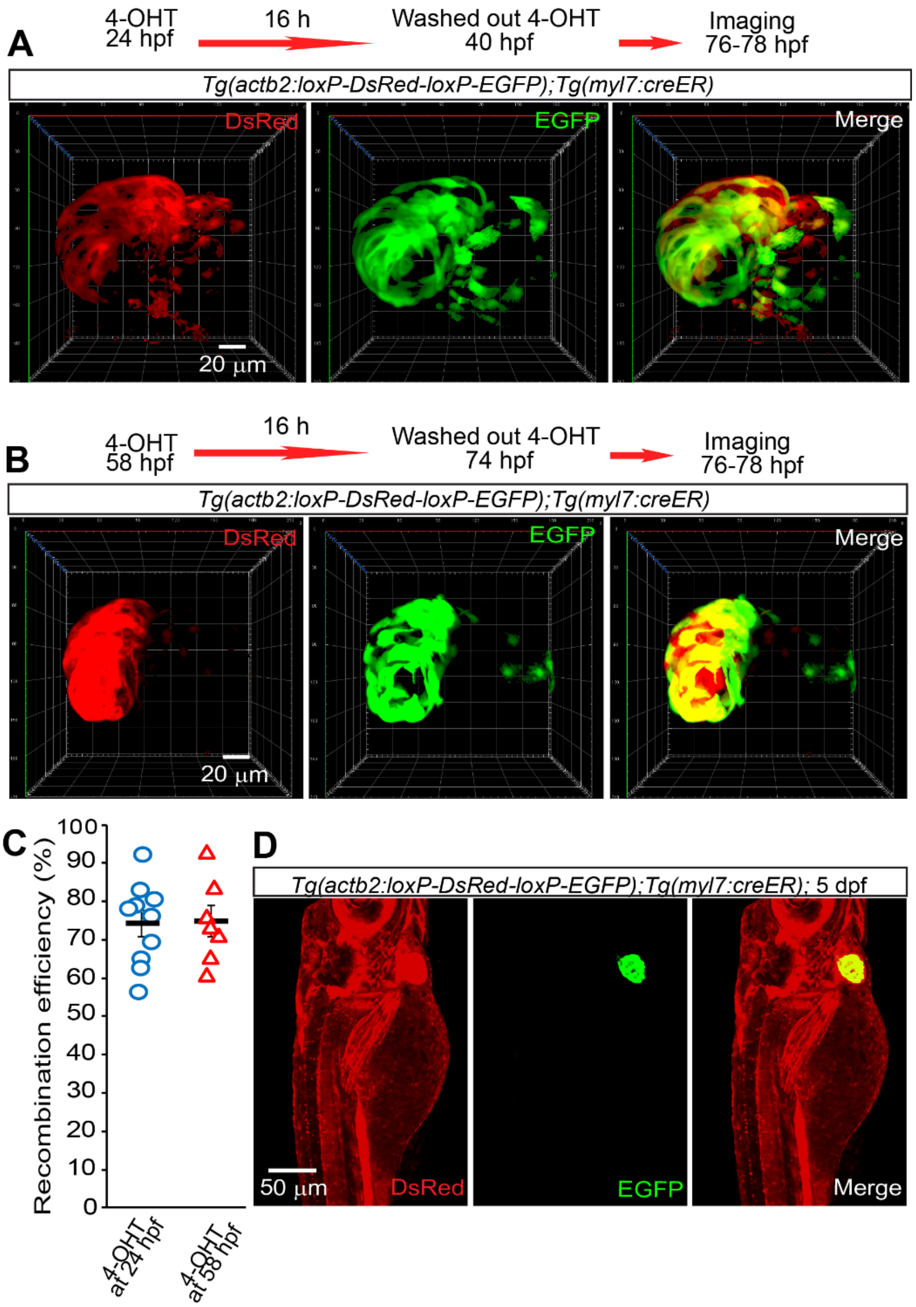

Supplementary Figure 3. Recombination efficiency and tissue specificity of *Tg(myl7:CreER)*. *Tg(actb2:loxP-DsRed-loxP-EGFP);Tg(myl7:creER)* embryos were treated with 4-OHT from 24 to 48 (**A**) or from 58 to 74 (**B**) hpf. Cre recombination-mediated EGFP (green) expression and non-recombined cells expressing DsRed (red) were assessed by live imaging from 76 to 78 hpf. Since it was not possible to count the number of cells due to the dense expression pattern of DsRed and EGFP, in order to quantify recombination efficiency we measured the area covered by EGFP expression (recombined cells) and the total cardiac ventricle area (combined EGFP and DsRed signals) in each Z-plane. **C**, Graph showing recombination efficiency calculated as percentage of GFP^+ area/total cardiac ventricle area. **D**, *myl7:creER* mediates myocardial-

specific recombination. 4-OHT treatment of *Tg(actb2:loxP-DsRed2-loxP-EGFP);Tg(myl7:creER)* embryos from 24 to 40 hpf resulted in EGFP (green) expression only in the myocardium, imaged at 5 dpf, despite ubiquitous expression of DsRed (red). 3D volume renderings (**A** and **B**) or maximum intensity projections (**D**) of 170 (**A**), 122 (**B**), 406 (**D**) μm thick confocal stacks are shown. **A** and **B** are ventral views, anterior up. **D** is lateral view, anterior up and dorsal to the left. Bars and error bars in **C** represent means±S.E.M. Each circle (n=10) and triangle (n=7) represents a heart. 10 (**A**), 7 (**B**), and 5 (**C**) animals were analyzed.

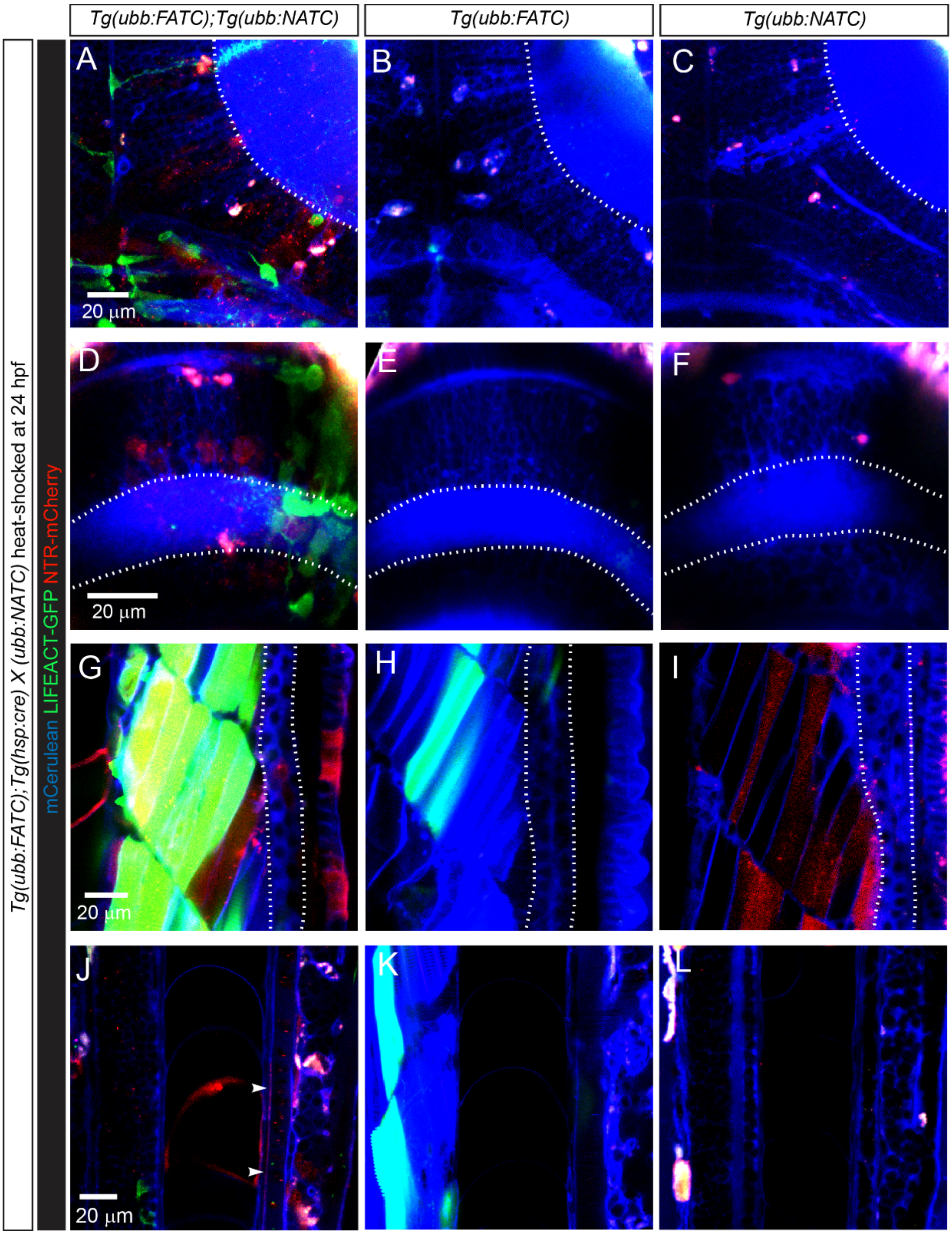

Supplementary Figure 4. Comparable expression patterns of FATC and NATC reporters in specific tissues. Embryos from crosses of *Tg(hsp:cre)*;*Tg(ubb:FATC)* to *Tg(hsp:cre)*;*Tg(ubb:NATC)* animals were heat-shocked at 24 hpf and sorted at 3 dpf as *Tg(ubb:FATC);Tg(ubb:NATC)* (**A**, **D**, **G**, and **J**), *Tg(ubb:FATC)* (**B**, **E**, **H**, and **K**), or *Tg(ubb:NATC)* (**C**, **F**, **I**, and **L**) based on LIFEACT-GFP (green) and NTR-mCherry (red) expression in skeletal muscles. Skeletal muscle (**H** and **K**), myocardium, and skin (see Fig. **S1**) are the only tissues showing LIFEACT-GFP or NTR-mCherry expression in the presence of *Tg(ubb:FATC)* or *Tg(ubb:NATC)* alone. Radial glial cells and neurons in the brain (**A**-**C**), and the retina (**D**-**F**), pronephric duct cells (**G**-**I**), and endothelial cells (**J**-**L**) expressed F/NATC reporter only in *Tg(ubb:FATC);Tg(ubb:NATC)* larvae (in which Cre recombination of both transgenes can generate GAL4 and UAS:LIFEACT-GFP, or UAS:NTR-mCherry, expression cassettes independently of ploidy number or fusion). All images are average or maximum intensity projections of 20 (**A**-**F**) and 4 (**G**-**L**) µm thick confocal stacks of 6 dpf larvae. White dashed lines mark tectal neuropil (**A**-**C**), inner plexiform layer (**D**-**F**), pronephros (**G**-**I**). White arrowheads (**J**) point to NATC^{+} cells in the dorsal aorta. Note that some autofluorescent cells are present in the brain (**A**-**C**), skin and gut (**J**-**L**). 6 *Tg(hsp:cre)*;*Tg(ubb:FATC)*, 9 *Tg(hsp:cre)*;*Tg(ubb:NATC),* and 8 *Tg(hsp:cre)*;*Tg(ubb:FATC); (ubb:FATC)* animals were examined.

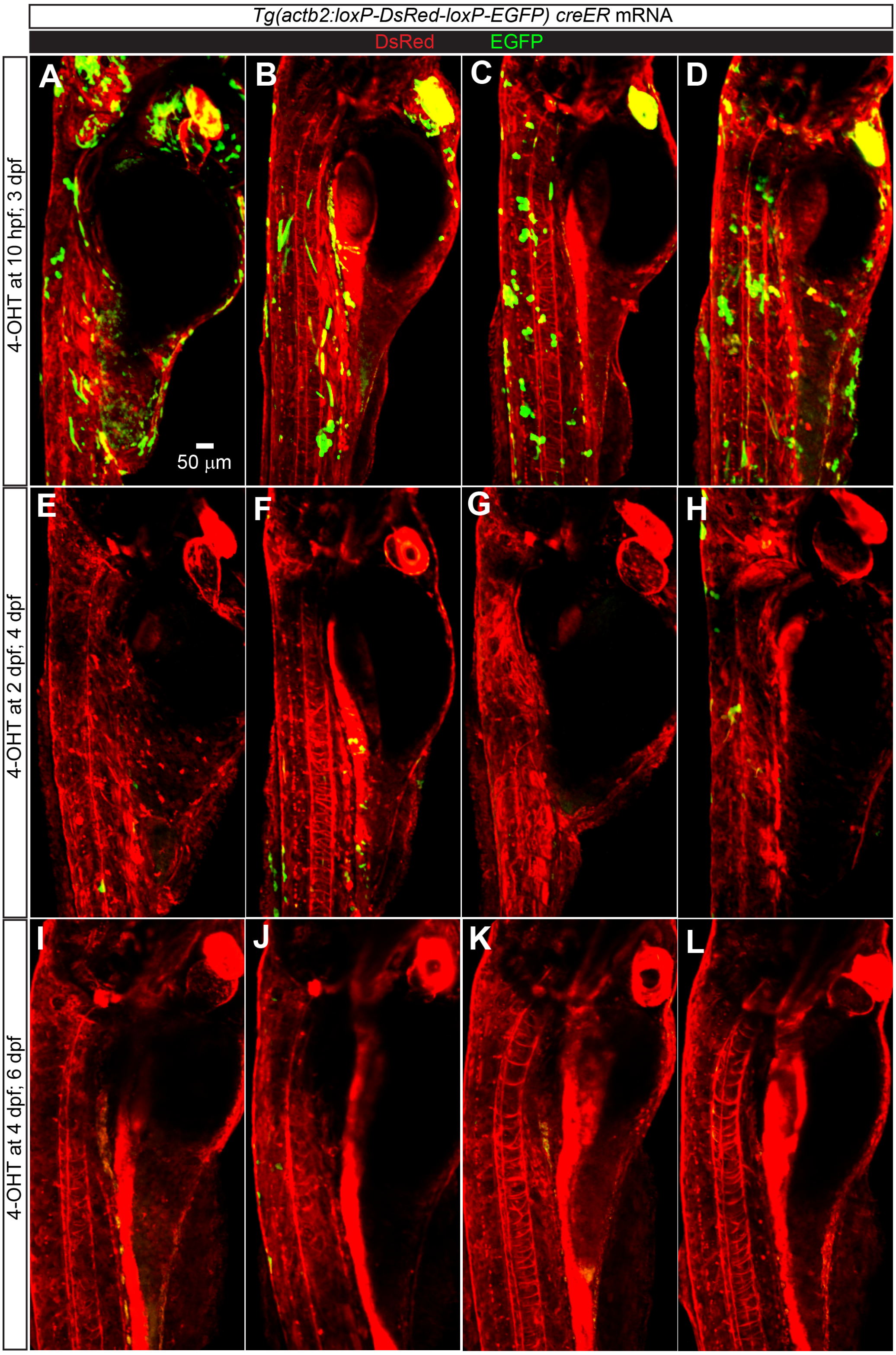

Supplementary Figure 5. Cre activity from injected *creER* mRNA appears to be lost by 4 days. *Tg(actb2:loxP-DsRed-loxP-EGFP)* embryos were injected with *creER* mRNA at the 1-4 cell-stage. Embryos and larvae were then subjected to an overnight treatment with 4-OHT at 10 hpf (**A-D**), 2 dpf (**E-H**), or 4 dpf (**I-L**). Images were acquired 48 hours after the 4-OHT pulse. Cre activity was detectable by EGFP expression (green). DsRed expression (red) persisted in cells

with no Cre activity. All images are maximum intensity projections of 200-350 μm thick confocal stacks and are shown in lateral views, dorsal to the left, anterior up. 6-10 animals were examined for each experimental condition.

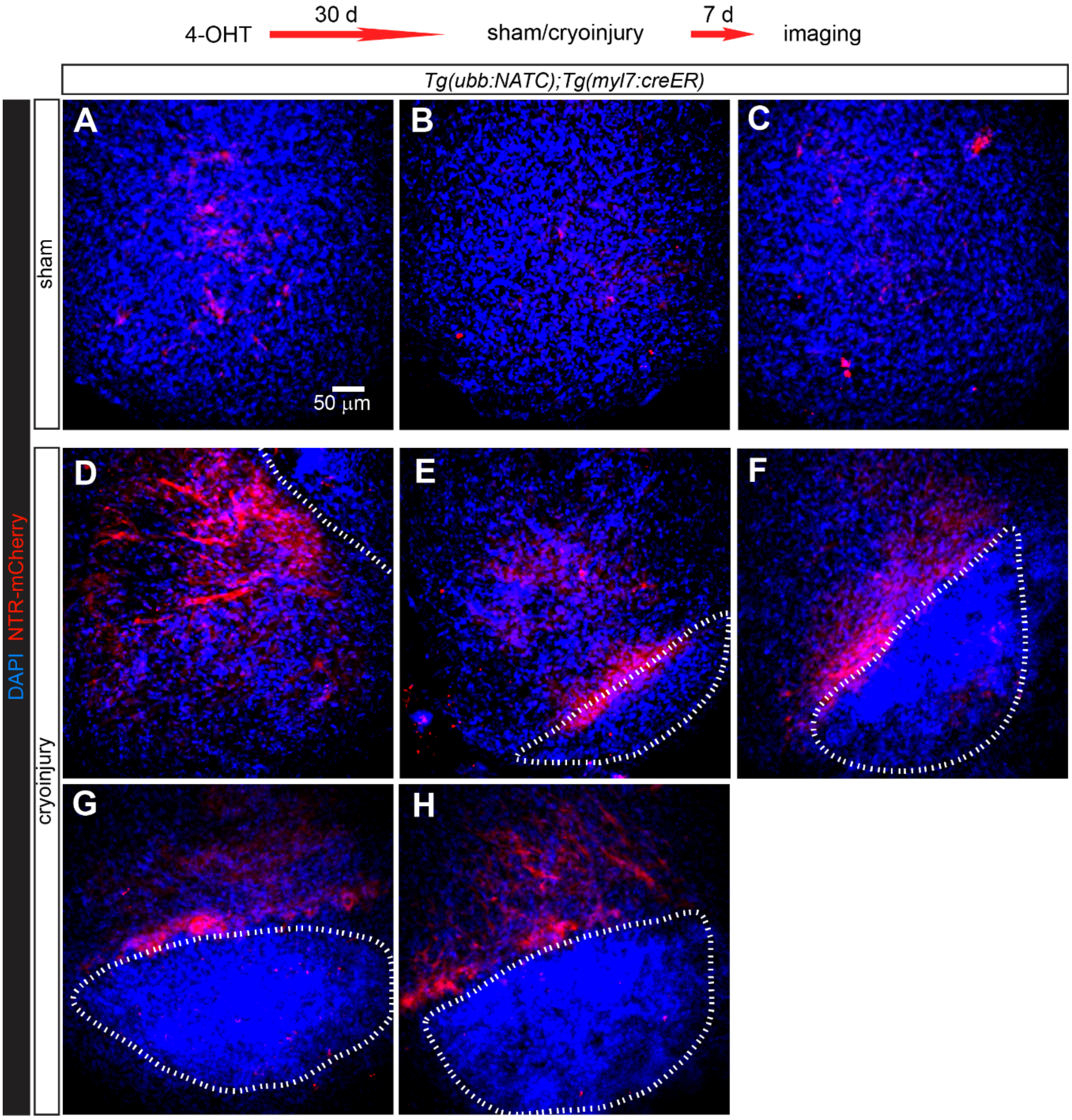

Supplementary Figure 6. Cardiac injury induces NATC reporter expression. *Tg(ubb:NATC);Tg(myl7:creER)* fish were 6 months old when cardiomyocyte-specific CreER was activated by intraperitoneal 4-OHT injection. 30 days later, the animals were subjected to cardiac cryoinjury or sham operation. NTR-mCherry expression (red) in sham-operated (**A**-**C**) and cryoinjured (**D**-**H**) hearts from sibling animals were visualized by immunostaining at 7 days post injury (dpi). DAPI (blue) nuclear staining shows disorganized cells in the damaged area, outlined by white dashed lines. Each image is an average intensity projection of an individual heart from a

200-300 µm thick confocal stack. A total of 4 sham and 6 cryoinjured hearts were analyzed and are shown here and in Fig. **6A**.

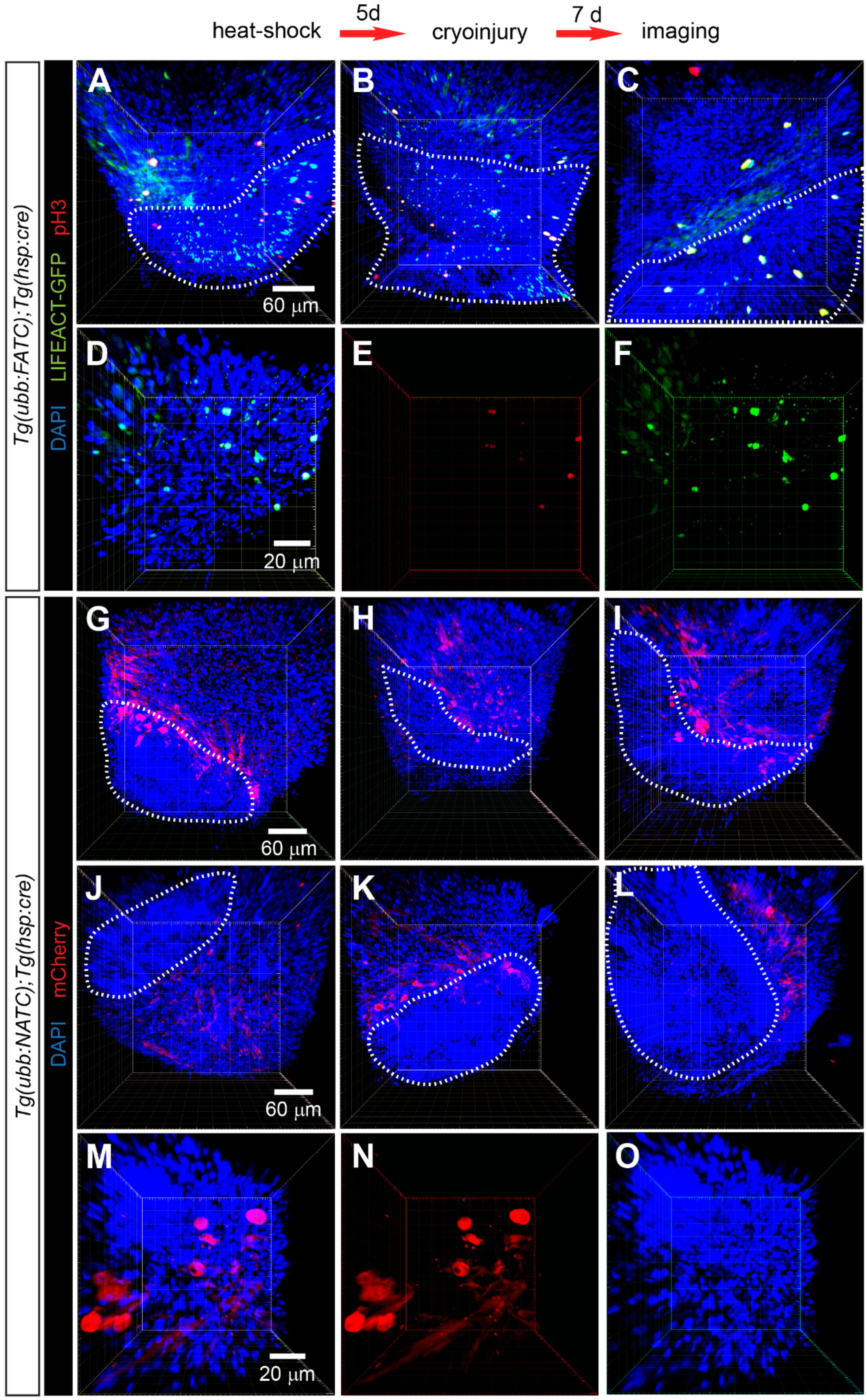

Supplementary Figure 7. F/NATC reporter expression correlates with cell proliferation during adult heart regeneration. A-F, FATC$^+$ cells proliferate during cardiac regeneration. *Tg(ubb:FATC);Tg(hsp:cre)* fish (20 months old) were heat-shocked to induce *cre* expression, followed by cardiac cryoinjury 5 days later. Activation of FATC reporter, visualized by LIFEACT-GFP expression (green), and mitotic activity, detected with phospho-Histone H3 immunostaining (pH3, red), in injured hearts was assessed at 7 dpi. Disorganized cells, visualized by DAPI (blue) staining, define the damaged area (white dashed lines). **G-L**, NATC-activated cells localized mainly adjacent to the damaged area. 12-month-old *Tg(ubb:NATC);Tg(hsp:cre)* fish were heat-shocked to induce *cre* expression 5 days prior to cardiac cryoinjury. NTR-mCherry expression (red) was visualized by immunostaining at 7 dpi. DAPI (blue) nuclear staining shows disorganized cardiac cells in the injured area, outlined by white dashed lines. **M-O**, NTR-mCherry$^+$ cells exhibit an atypical morphology that resembles immature/dedifferentiated cardiomyocytes. Each image is from an individual heart shown as a 3D volume rendering of a 100-400 μm thick confocal stack. 3 *Tg(ubb:FATC);Tg(hsp:cre)* and 6 *Tg(ubb:NATC);Tg(hsp:cre)* animals were examined.

Sawamiphak et al.

Figure S8

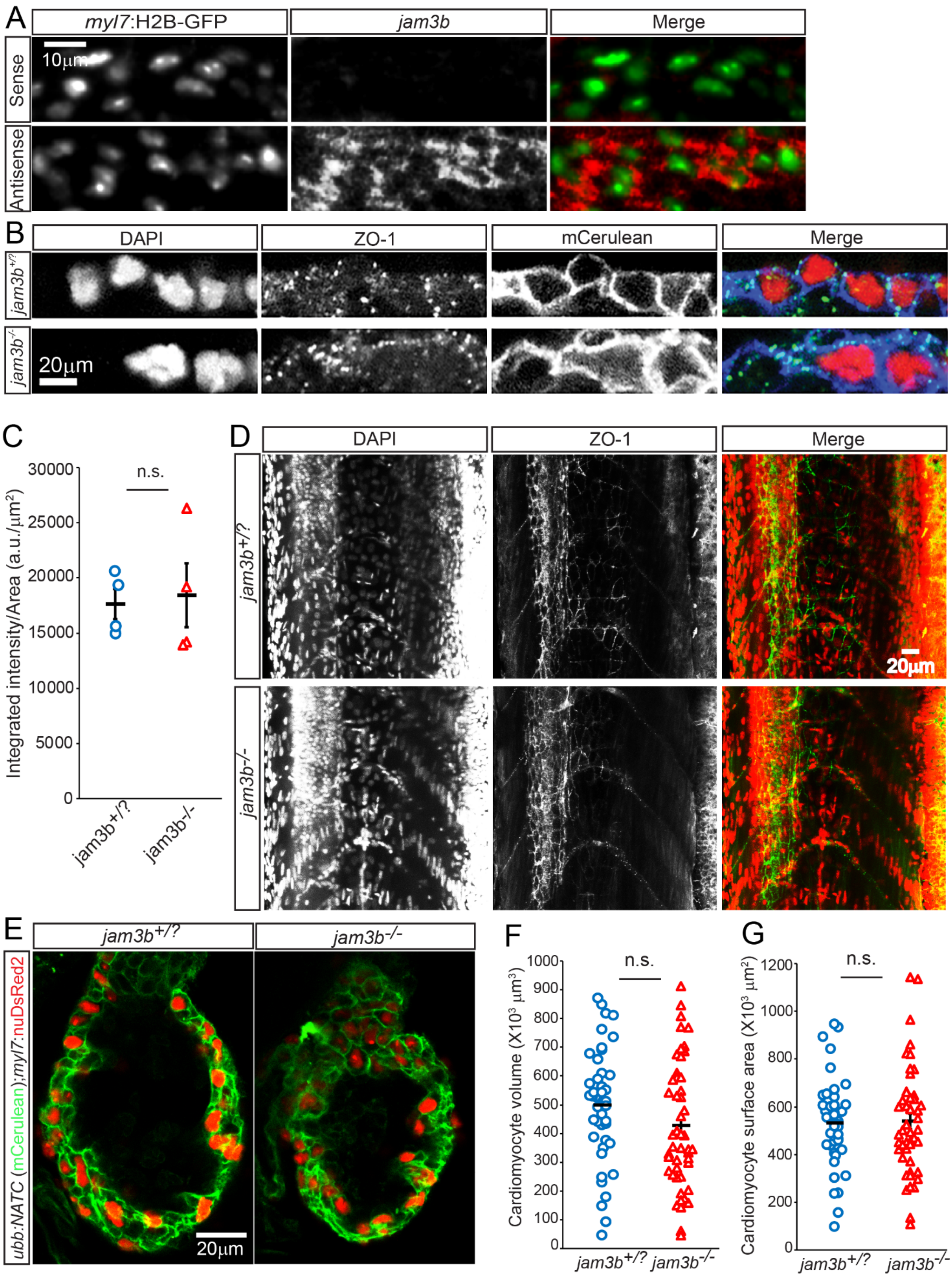

Supplementary Figure 8. Unaltered cardiomyocyte adhesion and morphology in *jam3b* mutants. **A**, *In situ* hybridization detected the expression of *jam3b* (red) in cardiomyocytes, labeled by *myl7*:H2B-GFP expression (green nuclei). **B**, Expression levels of the tight junction-associated protein ZO-1 at the plasma membrane of $jam3b^{-/-}$ hearts were comparable to those of control animals ($jam3b^{+/?}$ = $jam3b^{+/+}$ or $jam3b^{+/-}$). 3 dpf animals from $jam3b^{+/-}$*;Tg(ubb:NATC)* X $jam3b^{+/-}$ crosses were processed for whole mount immunostaining for ZO-1 (green) and GFP (detecting membrane Cerulean, blue). DAPI labeled nuclei are shown in red. **C**, Quantification of ZO-1 expression levels based on integrated intensity of the ZO-1 signals per plasma membrane area of individual cardiac ventricle. **D**, Jam3b deficiency does not appear to affect tight junction formation in epithelial or endothelial tissues. Expression and localization of ZO-1 (green), detected at 3 dpf by immunofluorescence, in $jam3b^{-/-}$ larvae appear identical to those in control $jam3^{+/?}$ siblings. DAPI staining (red) shows that skeletal myocytes are mononucleated in $jam3b^{-/-}$ fish, while they are mutinucleated in $jam3b^{+/?}$ siblings. **E-G**, Jam3b deficient animals show unaltered cardiomyocyte morphology. **E**, 5 dpf larvae from $jam3^{+/-}$*;Tg(ubb:NATC)* X $jam3^{+/-}$*;Tg(myl7:nuDsRed2)* crosses were immunostained to detect membrane Cerulean (green) and nuclear DsRed2 (red) expression. **F, G,** Graphs showing that cardiomyocyte volume (**F**) and surface area (**G**) were not altered in $jam3b^{-/-}$ larvae. Bars and error bars in **C**, **F**, and **G** represent means±SEM. n.s. not significant. Images are maximum or average intensity projections of 4 (**A**, **B**, and **E**), and 20 (**D**) µm thick confocal stacks shown in lateral views. 6-12 (**A**), 4 (**B** and **C**), 6-8 (**D**) larvae and 44 (**F**) or 49 (**G**) cardiomyocytes were examined.

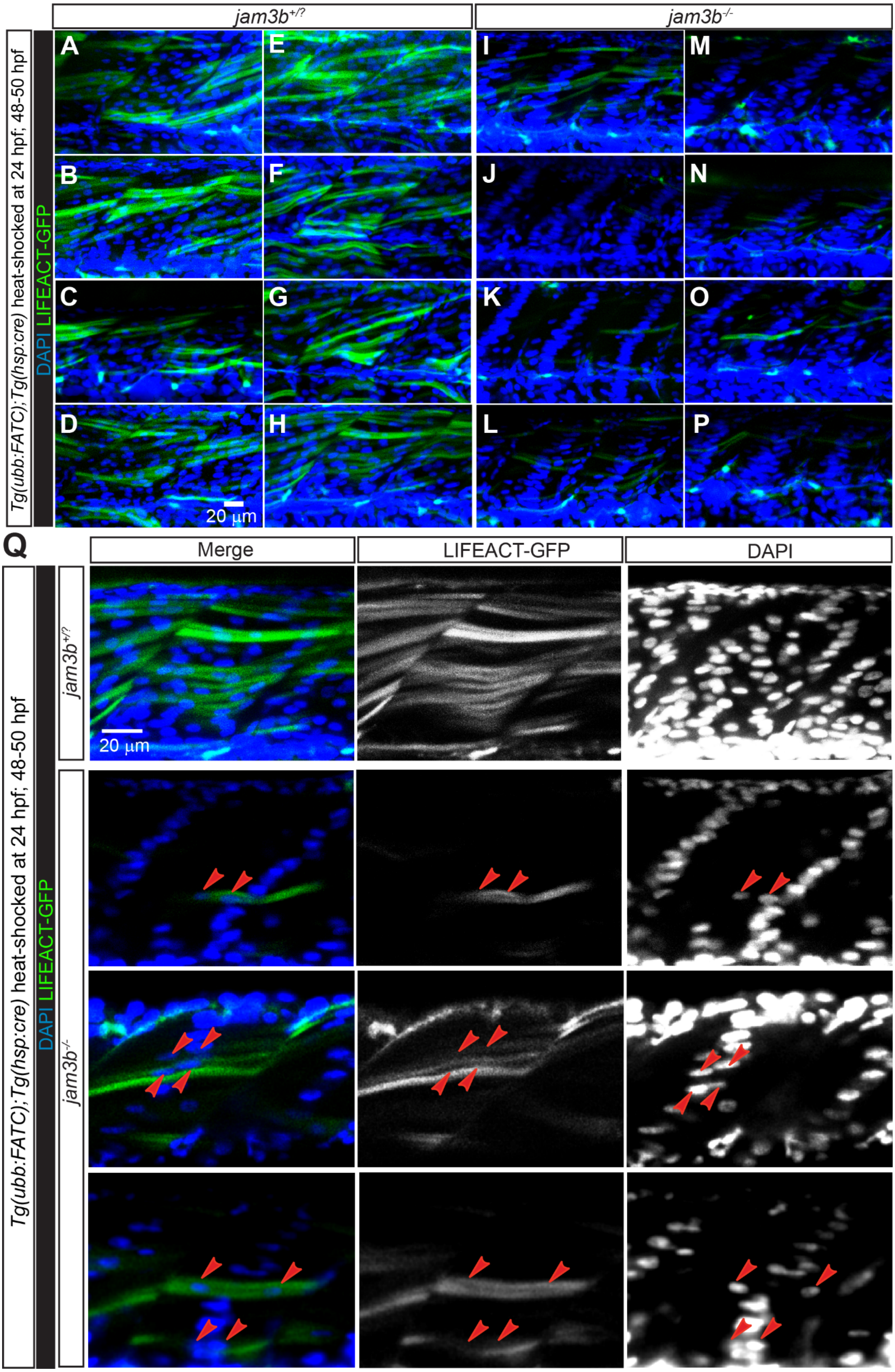

jam3b+/?
jam3b-/-
Tg(ubb:FATC);Tg(hsp:cre) heat-shocked at 24 hpf; 48-50 hpf
DAPI LIFEACT-GFP
A
B
C
D
E
F
G
H
I
J
K
L
M
N
O
P
20 µm
Q
Merge
LIFEACT-GFP
DAPI
jam3b+/?
jam3b-/-
20 µm
Tg(ubb:FATC);Tg(hsp:cre) heat-shocked at 24 hpf; 48-50 hpf
DAPI LIFEACT-GFP

Supplementary Figure 9. Jam3b deficiency impedes FATC activation in skeletal muscles. **A-P**, Embryos from crossing *jam3b*$^{+/-}$*;Tg(ubb:FATC)* fish to *jam3*$^{+/-}$*;Tg(hsp:cre)* fish were heat-shocked at 24 hpf. At 48-50 hpf, skeletal muscles in control animals (**A-H**, *jam3b*$^{+/?}$*=jam3b*$^{+/+}$ or *jam3b*$^{+/-}$) were multinucleated, as visualized by DAPI staining (blue), and showing LIFEACT-GFP (green) expression. By contrast, in *jam3b* mutants (**I-P**, *jam3b*$^{-/-}$), most muscles were mononucleated and their nuclei centrally positioned within each myotome. Correspondingly, the numbers of LIFEACT-GFP^{+} skeletal myocytes were reduced. **Q**, A few LIFEACT-GFP^{+} (green) myocytes detected in *jam3b*$^{-/-}$ embryos were multinucleated, as shown by DAPI staining (blue). Arrowheads point to nuclei of LIFEACT-GFP^{+} cells. Images are maximum intensity projections from 17.5-25 (**A-P**) and 10 (**Q**) μm thick confocal stacks of individual animals and shown in lateral views, dorsal to the left, anterior up. 10-12 embryos of each genotypes were examined.

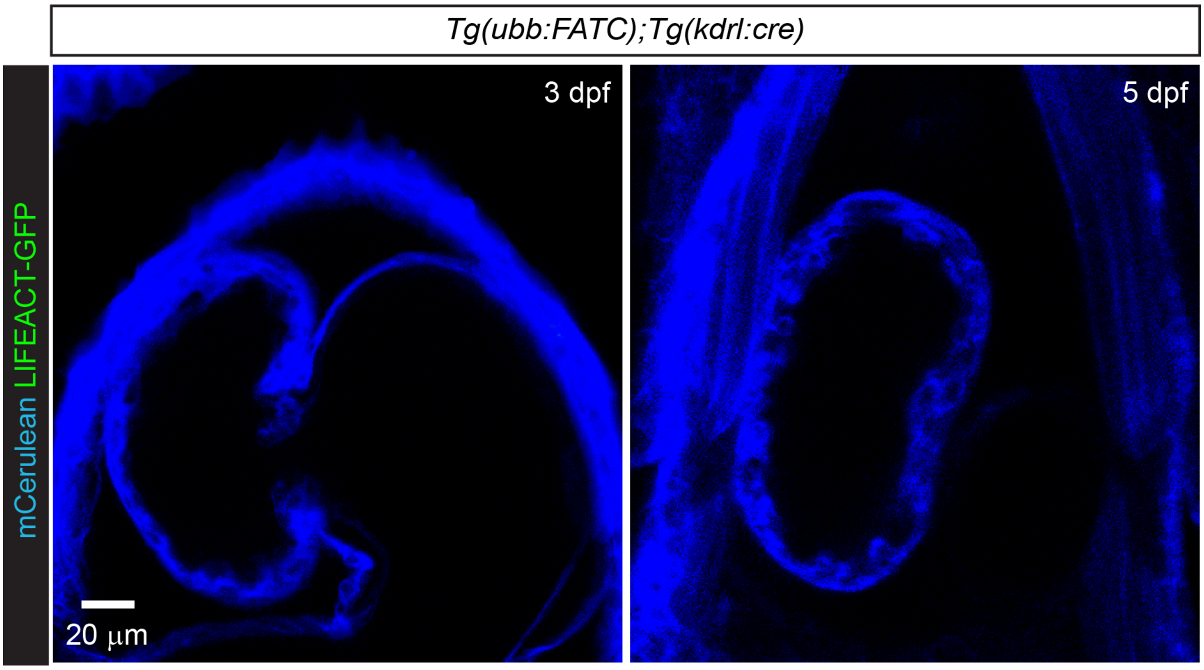

Supplementary Figure 10. Endothelial lineage cells are not involved in cardiomyocyte FATC reporter expression. *Tg(ubb:FATC)* ;*Tg(kdrl:cre)* animals in which *cre* was constitutively expressed in endothelial and hematopoietic progenitor cells showed no LIFEACT-GFP^{+} (green) cells in the myocardium. mCerulean expression (blue) confirmed the presence of the *ubb:FATC* transgene. LIFEACT-GFP induction was assessed in 3 and 5 dpf animals. Images are average intensity projections of 12 μm thick confocal stacks shown in ventral views, anterior up. 12 animals were examined at each developmental stage.
