# Supplementary material for: Transient cardiomyocyte fusion regulates cardiac development in zebrafish
Source: Nat Commun. 2017 Nov 15;8:1525. doi: 10.1038/s41467-017-01555-8 (PMC5688123; doi:10.1038/s41467-017-01555-8)
Supplement: Supplementary file 2 — Description of Additional Supplementary Files [file 41467_2017_1555_MOESM2_ESM.docx]

**Description of Additional Supplementary Files**

File Name: Supplementary Movie 1

Description: Cardiomyocytes undergo disruption and re-establishment of plasma membranes. A *tnnt2* MO-injected *Tg(myl7:MKATE-CCAX)*;*Tg(myl7:H2B-GFP)* heart was imaged starting at 54 hpf for 16 hours. Images are 3D-volume renderings from 60 µmthick confocal stacks, showing the luminal side of the compact myocardial wall. MKATECCAX (red) and H2B-GFP (green) labeled cardiomyocyte membranes and nuclei, respectively. Cardiomyocytes 1 and 2 initially seen as a binucleated cardiomyocyte establish a membrane border (white arrows). The border between cardiomyocytes 1 and 3 (white arrowheads) was transiently disrupted. Frames were taken every 20 mins. Frame rate is 3 frames/s. Time stamp is shown as hour:minute. A representative heart from a total of 6 hearts examined, all showing examples of cytoplasmic continuum between cardiomyocytes, is shown.
